# 2-Hydroxyglutarate in onco-pathway: a metabolic driver of proto-oncogene activation

**DOI:** 10.3389/fonc.2026.1791517

**Published:** 2026-04-30

**Authors:** Masthan Thamim, Debdas Dhabal, M. M. Balamurali, Krishnan Thirumoorthy

**Affiliations:** 1 Department of Chemistry, School of Advanced Sciences, Vellore Institute of Technology, Vellore, Tamil Nadu, India; 2 Department of Chemistry, Indian Institute of Technology Guwahati, Guwahati, Assam, India; 3 Department of Chemistry, School of Advanced Sciences, Vellore Institute of Technology, Chennai, Tamil Nadu, India; 4 School of Computer Science and Engineering, Vellore Institute of Technology, Vellore, Tamil Nadu, India

**Keywords:** 2-hydroxyglutarate, cancers, chirality, epigenetics, hypoxia, oncometabolite

## Abstract

Metabolic alteration has emerged as a hallmark of cancer, reshaping cellular energetics and biosynthetic pathways through enzyme denaturation, mutations, acidic pH, and hypoxia to sustain uncontrolled proliferation. Under these conditions, enzymes exhibit aberrant activity, disrupting wild-type processes and generating small chiral or achiral metabolites that often function as oncogene activators. Among these oncometabolites, 2HG has gained prominence due to its chiral forms, *D*-2HG and *L*-2HG, which structurally mimic α-KG and competitively inhibit α-KG-dependent dioxygenases. This enantioselective interference perturbs epigenetic regulation, redox balance, DNA repair, and cell signaling, thereby orchestrating tumor initiation and progression. This review provides an integrated overview of the factors driving chiral discrimination of 2HG, its contribution to tumor heterogeneity, and its influence on therapy resistance. By unravelling the molecular intricacies of 2HG enantiomers, this work highlights their significance as biomarkers and therapeutic targets, offering new avenues to disrupt cancer signaling and improve patient outcomes.

## Graphical Abstract

**Figure:**
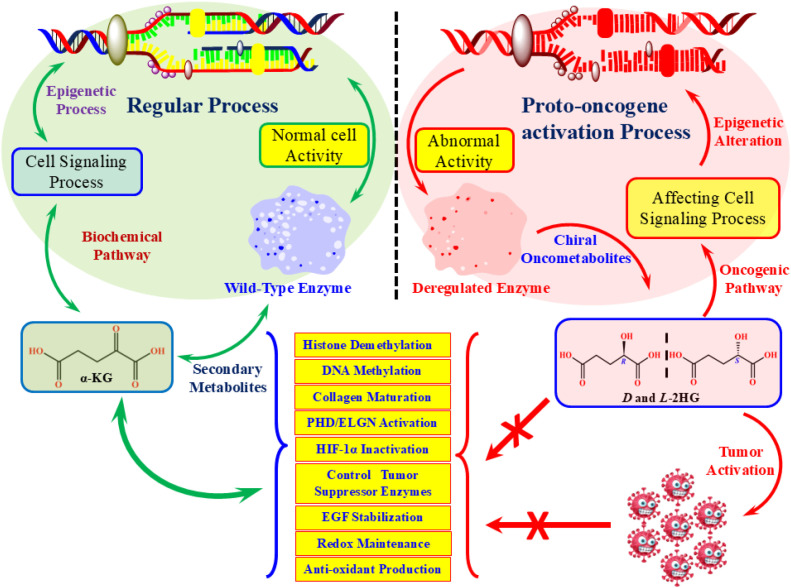

## 1 Introduction

Cancer is a group of aggressive diseases and a leading cause of mortality across all living organisms, typically arising from genetic and metabolic disorders. Specific to human beings, it is the second most common cause of death worldwide, with 18.1 million new cases and 9.6 million deaths reported in 2018, equivalent to one in every six deaths being attributed to cancer. Alarmingly, 70% of cancer-related deaths occur in low and middle-income countries. The global economic burden of cancer was estimated at 1.16 trillion USD in 2010 and is projected to escalate to nearly 47 trillion USD by 2030 (www.who.int). This devastating human and economic impact underscores the urgent need for a deeper study of cancer biology in addressing this global health crisis. Genetic changes drive the uncontrolled growth of cancer cells, leading to the formation of malignant tumors that rapidly spread throughout the body. Unlike normal cells, which follow regulated pathways and cease proliferating once they reach their local environment. But cancer cells disregard structural boundaries, aggressively infiltrating neighboring tissues and disrupting normal cellular function. This uncontrolled proliferation disrupts normal cell function and ultimately leads to cell death. Despite decades of research, more than 100 types of cancer have been described, yet the underlying causes and molecular mechanisms remain incompletely understood. Approximately 1/3 of cancer deaths are linked to obesity, poor diet, physical inactivity, aging, alcohol and tobacco use, and other lifestyle-related factors. In addition, non-modifiable elements, such as inherited genetic mutations, DNA repair defects, hormonal imbalances, and immune dysfunction, further contribute to cancer risk. Beyond these intrinsic drivers, three major external factors play a leading role in cancer initiation: (i) physical factors such as ultraviolet and ionizing radiation, (ii) chemical factors including carcinogens, food additives, and environmental pollutants, and (iii) biological factors such as infections from parasites, Helicobacter pylori, and oncogenic viruses including HIV, HPV, Epstein-Barr, and Hepatitis B and C. Among all risk factors, tobacco use is particularly significant, contributing to approximately 22% of global cancer deaths. These diverse factors interact with the human genome to produce constant mutational stress, which disrupts cellular processes and initiates the development of tumors (1). Once activated, cancer cells target the lymphatic system, an immune defense network comprising small kidney-shaped organs, distributed throughout the body. Impairment of lymph node function allows malignant cells to spread through the bloodstream, seeding distant organs and driving metastasis (2). Consequently, the overall survival rate for cancer patients remains poor (1).

At the biochemical level, cancer cells play a central role in metabolic reprogramming, adapting their metabolism to sustain uncontrolled growth (3). These adaptations affect intracellular redox reactions, disturb the balance between anabolism (the biosynthesis of essential compounds) and catabolism (energy production), and redirect both oxidative (mitochondrial) and non-oxidative (cytoplasmic) processes. Broadly, cellular metabolism encompasses three key processes: (i) energy production via adenosine triphosphate (ATP), (ii) anabolic synthesis through glycolytic intermediates, and (iii) the generation of small metabolites. These small metabolites act as fuels, signaling molecules, and enzyme substrates involved in epigenetic and gene regulations (4). Cancer cells, often described as “metabolic parasites,” take over these processes to support their rapid proliferation. A hallmark example is the Warburg effect, in which glucose is preferentially converted to lactate (LA) even in the presence of oxygen (5). In normal cells, oxygen availability favors oxidative phosphorylation for efficient ATP production. In contrast, cancer cells exploit oxygen yet deliberately engage in anaerobic-like glycolysis, producing LA through less energy-efficient pathways. This metabolic switch has been widely recognized as a hallmark of cancer and contributes to tumor progression by disrupting glycolytic flux and biosynthetic intermediates (6).

Beyond glycolysis, mitochondrial respiration is also implicated in cancer progression. Where it’s being sensitive to nutrient depletion, enzymatic deregulation, hypoxia, pH fluctuations, and the accumulation of oncometabolites (7). For example, the embryonic pyruvate kinase isoform M2 (PKM2), which is typically downregulated after birth, is frequently re-expressed in cancers. PKM2 overexpression drives LA production, thereby sustaining tumorigenesis (8). Such events arise from persistent genetic mutations that deregulate enzymatic function (9). These mutations generate deviant products, such as mutant enzymes or inactive proteins, which disrupt normal pathways and promote tumorigenesis by accumulating oncometabolites. Oncometabolites contribute to epigenetic alterations, including DNA and histone methylation. These abnormalities inhibit regulatory enzymes and impair DNA repair, making their augmentation a decisive factor in cell fate and tumorigenesis (10).

Several conditions amplify oncometabolite conglomeration. For example, during glycolysis and oxidative phosphorylation, excessive LA and H^+^ ions lower intracellular pH. Similarly, in mitochondrial respiration, the excess CO_2_ is hydrated by extracellular carbonic enzyme, which may crucially convert HCO_3_^-^ and H^+^ that further acidify the tumor microenvironment (11). Cancer cells also produce LA at far higher levels than normal cells, prolonging the survival of cancer metabolites. Altered pathways give rise to small molecules with oncogenic properties, collectively termed oncometabolites. This, oncometabolites activate oncogenic signaling via stimulating reactive oxygen species (ROS) through various cellular tracks such as the pentose phosphate pathway (PPP), phosphatidylinositol 3-kinase (PI3K), and mammalian target of rapamycin (mTOR) (9). Elevated ROS damages proteins, lipids, and nucleic acids, reducing cellular viability and exacerbating cancer progression (12). So, the study of oncometabolites is based solely on enzyme activity and function. Yet the catalytic role of enzymes is an extensive component that involves an abnormal mechanism to yield cancer metabolites. This metabolite has been considered as a biomarker to diagnose cancer growth in the early stages and to give fruitful information about the root causes. This information may lead to the development of clinical trials and stopping tumor recurrence after treatments, as they improve the cancer patient survival rate (10). Importantly, these metabolites can exist in both chiral and achiral forms (13, 14).

At this juncture, the role of chirality becomes essential. Chiral selectivity underpins thousands of biological processes, including enzyme-substrate catalysis, redox reactions, signal transduction, control of amino acid synthesis and tRNA folding, ribosomal protein formation, nucleic acid identification, transcription and translation, metabolite interactions with cell signaling pathways, and other activities (15, 16). These reactions are based on a different level of structural hierarchy, involving chiral molecules that aggregate chiral products (17). Notably, the small molecular weight of chiral molecules has been projected as antibiotics, activators, reaction controllers, chemotherapeutic agents, cell signaling ligands, and substrates in many enzymatic reactions (18, 19). The existence of chirality influences enzyme-catalyzed reactions by modulating the binding specificity of receptors and proteins, including those involved in oncogenesis (20). The primary objective of this review is to elucidate the pathways, mechanisms, and reactivity of chiral metabolites in biotransformation processes related to cancer proliferation. In this approach, the end products of chiral metabolites resulting from cellular function, particularly altered enzymatic function, have been scrutinized as a spectacle key constituent in ongoing cancer research. This function can be validated through various experimental analyses, including enzymatic assays, metabolite profiling using systems biology, and analytical instrumentation.

## 2 Factors that catalyze chiral discrimination of 2-hydroxyglutarate

Our primary focus in this review is the study of aberrant growth of 2-hydroxyglutarate (2HG) (C_5_H_8_O_5_; molecular mass 148.11 g/mol), which arises from diverse metabolic disruptions that redirect normal cell signaling processes. Notably, 2HG is a well-documented oncometabolite that exists as two enantiomers (*D* and *L* forms) and is widely reported in many cancers (21). Chiral discrimination of 2HG is not incidental but is driven by specific biochemical conditions that alter enzyme fidelity, substrate specificity, and repair efficiency, which yield cancer cell progression and other disorders. In this direction, the present work explicitly addresses four major factors underlying this phenomenon: enzyme malfunction, hypoxia, metabolic acidosis, and impairment of repair and rewiring mechanisms. Mutations or deregulations in metabolic enzymes introduce neomorphic activities that favor the production of *D*-2HG. Hypoxic environments stabilize hypoxia-inducible factors, which reprogram metabolism, thereby favoring *L*-2HG synthesis. Acidic and pH fluctuations within the tumor microenvironment further modulate enzyme conformation and substrate preference, thereby reinforcing the formation of abnormal oncometabolites. Finally, disruption of detoxifying enzymes impairs cellular repair systems, preventing the clearance of accumulated 2HG. The following subsections will address in depth these factors under the context of (2.1) enzyme malfunction, (2.2) hypoxia, (2.3) metabolic acidosis, and (2.4) repair and rewiring impairment. This comprehensive information is tailored to how these factors converge to drive the enantioselective production and persistence of 2HG in cancer.

### 2.1 Catalytic role and malfunction of enzyme activity

Enzymes are catalytic proteins that mediate nearly all biological processes necessary for life. Their catalytic efficiency is far greater than that of non-biological catalysts, enabling them to facilitate more than 5,000 biochemical reactions, including transcription, translation, protein synthesis, redox reactions for energy production, antibiotic biosynthesis, immune response, and many others (22). Among these, energy production is significant, as it underpins cellular respiration. The Krebs cycle, also known as the tricarboxylic acid (TCA) cycle, represents a central hub of cellular respiration and redox reactions, tightly coupled to the electron transport chain (ETC). Beyond generating ATP, it also produces a wide range of biomolecules, including fatty acids, steroids, and cholesterol for regulatory processes, as well as amino acids, purines, and pyrimidines for protein, DNA, and RNA synthesis (23). Enzymes within the TCA cycle play indispensable roles in maintaining cellular homeostasis. Malfunction of these enzymes can result in abnormal activity, leading to the generation of chiral or achiral secondary metabolites that aberrantly activate oncogenic pathways (10, 13). The following section explicitly highlights how mutations in key metabolic enzymes promote the formation of chiral oncometabolites and explores the mechanistic connections between these metabolites, cancer cell progression, and related disorders.

#### 2.1.1 Isocitrate dehydrogenase (IDH1/2)

Among metabolic enzymes, isocitrate dehydrogenases (IDHs) are particularly important. They catalyze the oxidative decarboxylation of isocitrate (ICT). They are central to major cellular processes, including mitochondrial oxidative phosphorylation, glutamate (GLU) metabolism, lipogenesis, glucose sensing, and redox balance regulation. The IDH family consists of three isoforms: IDH1, IDH2, and IDH3. IDH1 and IDH2 are structurally similar (≥70% homology), homodimeric, and nicotinamide adenine dinucleotide phosphate (NADP^+^) dependent enzymes, yet they function in distinct subcellular compartments (24). IDH1, located in the cytosol and peroxisome, comprises 414 amino acids with a molecular mass of 46.659 kDa and is encoded by the gene at locus 2q33.3 (www.genecards.org). Structurally, it consists of three domains: the large domain (residues 1-103 and 286-414), the small domain (residues 104-136 and 186-285), and the clasp domain (residues 137-185). Its active site lies at the interface of the large and small domains, accommodating NADP^+^, ICT, and Mn^2+^/Mg^2+^ metal-binding sites. During catalysis, the domains undergo conformational changes, shifting from an open to a quasi-open to a closed state, with the closed conformation representing the catalytically active enzyme (25, 26). Similarly, IDH2, located in mitochondria, consists of 452 amino acids with a molecular mass of 50.909 kDa and is encoded by the gene at locus 15q26.1. Its functional mechanism is comparable to IDH1, with large and small domains forming hydrophobic clefts that coordinate NADP^+^, ICT, and metal cofactors to achieve catalytic activity (26). Significantly, IDH2 is regulated by acetylation and deacetylation factors, where acetylation at Lys-256 reduces electrostatic repulsion, weakens ICT binding, and impairs α-ketoglutarate (α-KG) production, thereby compromising enzymatic activity (27). In contrast, IDH3 is a heterotetrameric, and nicotinamide adenine dinucleotide (NAD^+^) dependent mitochondrial enzyme composed of three subunits, IDH3A (366 amino acids, 39.592 kDa; gene locus 15q25.1), IDH3B (385 amino acids, 42.184 kDa; gene locus 20p13), and IDH3G (393 amino acids, 42.974 kDa; gene locus Xq28) arranged in a 2:1:1 ratio (25, 28). Unlike IDH1/2, IDH3 catalyzes an irreversible oxidative decarboxylation of ICT to α-KG, regulated allosterically by positive effectors (Ca^2+^, adenine dinucleotide phosphate (ADP), citrate) and negative effectors (ATP, NADH, NADPH). This positions IDH3 as a rate-limiting step in the TCA cycle, which is critical for mitochondrial energy production through NADH synthesis. It is imperative during embryonic development, where the availability of acetyl-CoA and α-KG governs epigenetic gene regulation and zygotic genome activation (29). The products of IDHs enzyme catalysis, α-KG, NAD(P)H, and CO_2_ are central to cellular metabolism. NAD^+^ is essential for DNA repair, post-translational modifications, and cell signaling, while the NAD^+^/NADH ratio is pivotal for maintaining energy homeostasis (30). NADPH, in turn, sustains antioxidant defenses via glutathione and thioredoxin pathways to mitigate oxidative stress and DNA damage, supporting lipid biosynthesis. Under hypoxic conditions, NADPH facilitates the reductive carboxylation of α-KG, supporting *de novo* lipid synthesis and cell survival by reducing ROS (31).

Deficiency or deregulation of IDH enzymes has profound physiological consequences. Loss of IDH1 functionality is associated with hepatic ROS accumulation, ultraviolet B (UVB)-induced phototoxicity, oxidative DNA damage, and impaired fatty acid synthesis, ultimately leading to increased lethality and inflammation (32). The reduced level of IDH1 is closely associated with the α-KG transamination step in gluconeogenesis, thereby lowering the rates of glucose, urea, and ammonia production in the liver. IDH1 overexpression, conversely, has been associated with obesity, fatty liver, and hyperlipidemia in transgenic mice (33). IDH2 actively maintains the mitochondrial redox balance to protect against mitochondrial dysfunction. Loss of IDH2 functionality disrupts mitochondrial redox homeostasis, leading to heart failure with cardiac hypertrophy, ischemia-reperfusion injury in the liver, and oxidative damage in kidney tubule cells (34). IDH2 also connects with pyruvate dehydrogenase (PDH), oxidative phosphorylation, and acetyl-CoA production, supporting lipid biosynthesis and maintaining citrate levels under hypoxic conditions (35). IDH3, with its unique regulation, governs mitochondrial energy production and epigenetic control through α-KG availability (29). Collectively, IDH enzymes play a crucial role in epigenetically regulating gene expression via α-KG-dependent dioxygenases. This downstream pathway robustly maintains cellular redox balance and enhances anaplerotic flux to support cell function by providing NADPH and precursor substrates for protein biosynthesis. Additionally, they modulate respiration and energy maintenance through the origination of NADH (36).

**Scheme 1:**
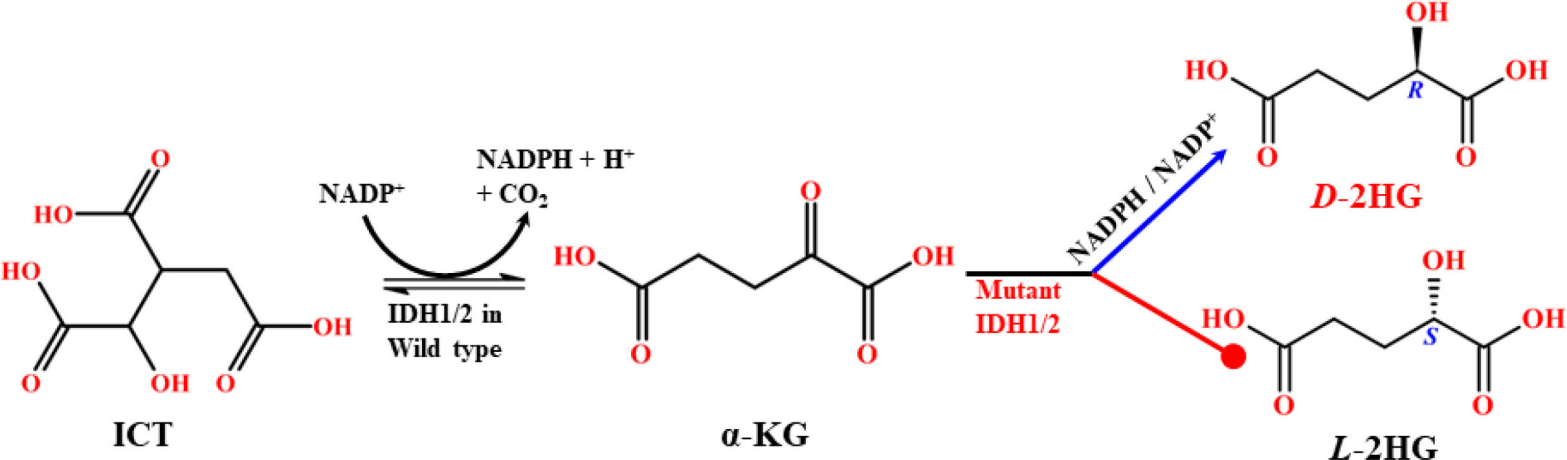
Mechanistic route of the wild-type and mutant type IDH1/2 reaction pathway.

The dysregulation of IDHs is closely linked to human diseases, most notably cancer. Mutations in IDH enzymes represent critical oncogenic events and are widely reported across various malignancies (37). IDH1/2 mutations, in particular, are strongly associated with tumorigenesis via chiral discrimination (13). Mutations in IDH3, especially IDH3A, have been implicated in lung and breast cancers at lower frequencies, as well as in non-malignant disorders such as night blindness and visual impairment (38). However, unlike IDH1/2, IDH3 mutations are not directly associated with chiral oncometabolite production. Numerous studies have documented IDH1/2 mutations in a spectrum of cancers. This includes, WHO grade II/III gliomas, grade IV glioblastoma (GBMs), acute myeloid leukemia (AML), intrahepatic cholangiocarcinoma, melanoma, angioimmunoblastic T cell lymphoma (AITLs), chondrosarcoma, sporadically in melanoma, medulloblastoma and prostate cancer and less frequently in the thyroid, stomach, breast, and pancreatic cancers and diseases including Ollier disease and Maffucci syndrome. IDH1/2 mutations occur in ≥80% of WHO grade II/III astrocytomas, oligodendrogliomas, glioblastomas, and oligoastrocytomas (24, 39, 40). These mutations are typically somatic, heterozygous, and missense, conferring a gain-of-function phenotype on wild-type enzymes. Interestingly, IDH1 and IDH2 mutations are highly recurrent at specific arginine residues, changing a different amino acid in the active sites. The particular mutation hotspots, namely R100Q, R132H, R132S, R132C, R132G, R132L in IDH1 and R140Q and R172K in IDH2, which dictate distinct catalytic properties that activate the oncogene pathway (41). Especially in IDH1, the most common mutation is R132H, which accounts for approximately 89% of mutations. In IDH2, the common mutations are R140Q and R172K, which play a primary role in driving extensive cancer progression. The remaining disparities are addressed infrequently and are represented as passenger mutations (42). Predominantly, the codon R132H in IDH1 mutations is associated with inactivating the tumor suppressor protein 53 (TP53), which plays a crucial role in the multicellular domain to prevent cancer formation (43). These mutations disrupt the normal oxidative decarboxylation of ICT to α-KG, conferring a neomorphic catalytic activity that reduces α-KG to *D*-2HG, consuming NADPH in the process (**Scheme 1**). Mutant IDH1/2 produce *D*-2HG at levels more than 100-fold higher than normal in tumor tissue studies (44), whereas wild-type enzymes do not. Notably, this gain-of-function is highly enantioselective, producing *D*-2HG, whereas *L*-2HG arises via different enzymatic defects (41). Both enantiomers are recognized as key biomarkers in multiple cancers (21).

#### 2.1.2 Hydroxyacid-oxoacid transhydrogenase

**Scheme 2:**
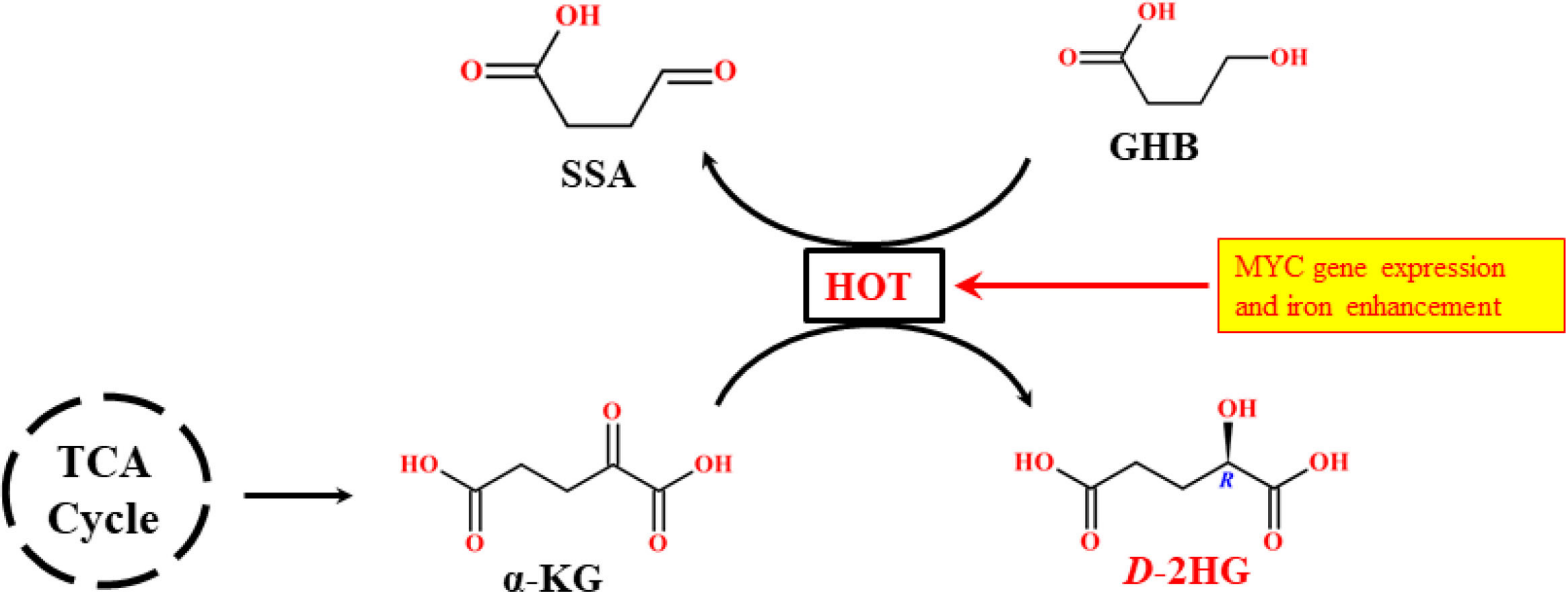
Mechanistic route of cofactor-independent hyperactivity of the HOT reaction pathway in *D*-2HG formation.

The enhancement of oncometabolite concentration can occur through multiple mechanisms, not limited to IDH mutations. Several other enzymes contribute significantly to elevating *D*-2HG in the cellular milieu. In this context, one of the most critical oxidoreductases is hydroxyacid-oxoacid transhydrogenase (HOT) (45), also known in its active form as alcohol dehydrogenase iron-containing protein 1 (ADHFE1). The iron-dependent HOT enzyme comprises 467 amino acids, has a molecular mass of 50.308 kDa, and is encoded on chromosome 8q13.1. As an iron-sulfur protein, HOT plays a vital role in regulating fatty acid and lipid synthesis, and in maintaining cellular redox balance (46). It is expressed at higher levels in the brain, heart, liver, and kidneys, and at lower levels in muscle and brown adipose tissue (47). As a mitochondrial enzyme, HOT can strongly influence *D*-2HG availability by coupling iron metabolism with *MYC* gene activation, thereby indirectly affecting IDH2 activity (48). The catalytic function of HOT is a mechanistically complex process. It performs a cofactor-independent reductive carboxylation of γ-hydroxybutyrate (GHB) into succinic semialdehyde (SSA), while simultaneously reducing α-KG into *D*-2HG (**Scheme 2**). It involves trans-hydrogenation, which mechanizes the process where GHB is oxidized while α-KG is reduced, producing SSA and *D*-2HG, respectively. Thus, α-KG and GHB act as dynamic substrates for HOT activity. Functionally, HOT reduces cellular α-KG levels, thereby interfering with wild-type IDH2 function (49).

The availability of HOT has been reported as a precursor to oncogene activation, especially in breast cancer, where it is upregulated through enhanced iron metabolism and *MYC* gene expression (50). Isotopomer analysis using knockdown of the repair enzyme D-2-hydroxyglutarate dehydrogenase (D2HGDH) has shown that elevated *D*-2HG concentrations in wild-type IDH2 breast cancer tissues are directly attributable to *MYC*-expressed HOT activity, with no genotype-phenotype correlation with other diseases (51). Hyperactivity of HOT thus augments *D*-2HG formation, strongly impacting patient survival. Collectively, these findings demonstrate that mitochondrial *D*-2HG driven by HOT affects wild-type IDH2 activity (52). Clinical studies further suggest that HOT hyperactivity may contribute to α-KG dehydrogenase deficiency. Elevated *D*-2HG has been detected in the body fluids of patients with this condition (53). The deficiency shifts the kinetic equilibrium of HOT toward *D*-2HG overproduction (48). Knockdown and inhibition studies confirm that reduced HOT activity lowers intracellular *D*-2HG, highlighting HOT as a crucial regulator of mitochondrial *D*-2HG pools. Importantly, experimental evidence demonstrates that in breast cancer tissues, *D*-2HG arises specifically from HOT activity, rather than from wild-type IDH enzymes (52). Consequently, millimolar concentrations of *D*-2HG detected in wild-type IDH2 breast cancer cells are attributed to *MYC-driven* HOT expression (51). Interestingly, hyperactive or mutated HOT has not been identified as a contributor to *D*-2HG formation in wild-type IDH glioblastoma patients, suggesting that HOT-arbitrated *D*-2HG generation is cancer-type specific. In breast cancer, HOT activity is highly correlated with *MYC* signaling, which enhances iron metabolism and drives *D*-2HG development in both body fluids and tumors. The resulting metabolic shift is striking, with HOT-mediated *D*-2HG levels increasing by 10-15 folds under hypoxic compared to aerobic conditions. Under hypoxia, HOT appears to reprogram metabolism by elevating ROS production, disrupting GLU metabolism, promoting epithelial-mesenchymal transition (EMT), and driving epigenetic modifications that support metastatic tumor development (54). Collectively, these findings highlight HOT as a critical, non-IDH source of *D*-2HG. Its hyperactivity induces metabolic variation via ROS generation, alterations in the GLU pathway, disruption of the TCA cycle, and broad epigenetic modifications. The resulting mesenchymal transition, cellular dedifferentiation, and oncogene activation strongly resemble the consequences of IDH mutations. Thus, HOT represents a parallel pathway of *D*-2HG driven oncogenesis with unique implications for breast cancer and potentially other malignancies.

#### 2.1.3 Phosphoglycerate dehydrogenase

Cancer cells reprogram their metabolism to consume disproportionately large amounts of glucose compared to wild-type cells. Instead of maximizing ATP production, they preferentially channel glucose into LA production, thereby sparing oxygen for biomass synthesis and survival pathways. This metabolic adaptation reprograms cellular processes to sustain rapid growth and proliferation (55). Disruptions in glycolysis, also known as the Embden-Meyerhof pathway (EMP), are central to tumor progression and oncogene activation, ultimately leading to uncontrolled cancer cell proliferation (56). A critical diversion within glycolysis is the serine synthesis pathway (SSP), which is phosphorylation-mediated and provides precursors for the non-essential amino acid *L*-serine, a metabolite vital for multiple cellular functions. The first step of SSP is catalyzed by phosphoglycerate dehydrogenase (PHGDH), which converts 3-phosphoglycerate (3-PG), derived from glucose, into 3-phosphohydroxypyruvate (3-PHP) with concomitant reduction of NAD^+^ to NADH (57). In this step, the enzyme undergoes a reversible reaction under standard conditions that is thermodynamically more favorable than the forward reaction. This reverse reaction in the downstream pathway potentially controls the level of serine synthesis. In the equilibrium state, less than 5% of substrate and product exist in the form of 3-PHP. But the forward reaction proceeds rapidly, converting 3-PHP to phosphoserine (PS) in reversible conditions via phosphoserine aminotransferase (PSAT1) and PS to serine by phosphoserine phosphatase (PSPH) and in an irreversible manner, thereby amplifying serine synthesis in SSP (56). Among these three enzymes, PHGDH is the rate-determining enzyme in the SSP route, playing a crucial role in activating serine biosynthesis. It diligently controls and diverts the glycolytic flux into serine synthesis to maintain cellular function in various aspects. Elevated intracellular serine concentrations are strongly linked to cancer development. Intriguingly, PHGDH has also been implicated in the formation of the oncometabolite *D*-2HG within the cellular milieu (57). Structurally, human PHGDH is a cytosolic enzyme that contains 533 amino acids with a molecular mass of 56.651 kDa, encoded at locus 1p12 (58).

**Scheme 3:**
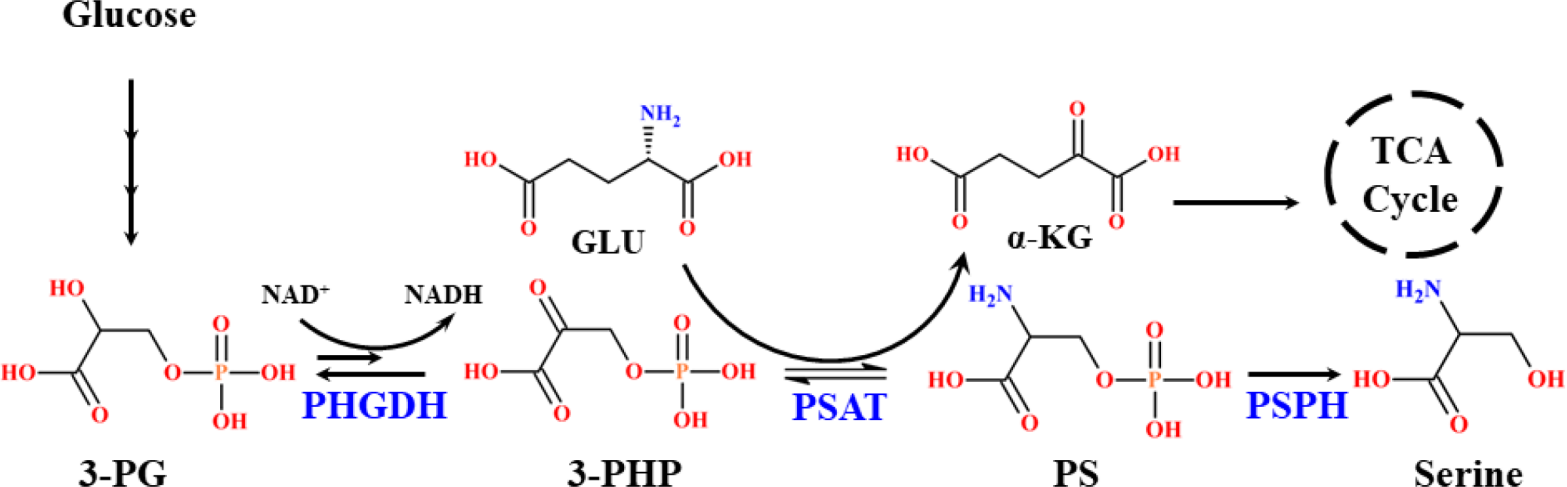
Mechanistic route of wild-type PHGDH reaction pathway.

**Scheme 4:**
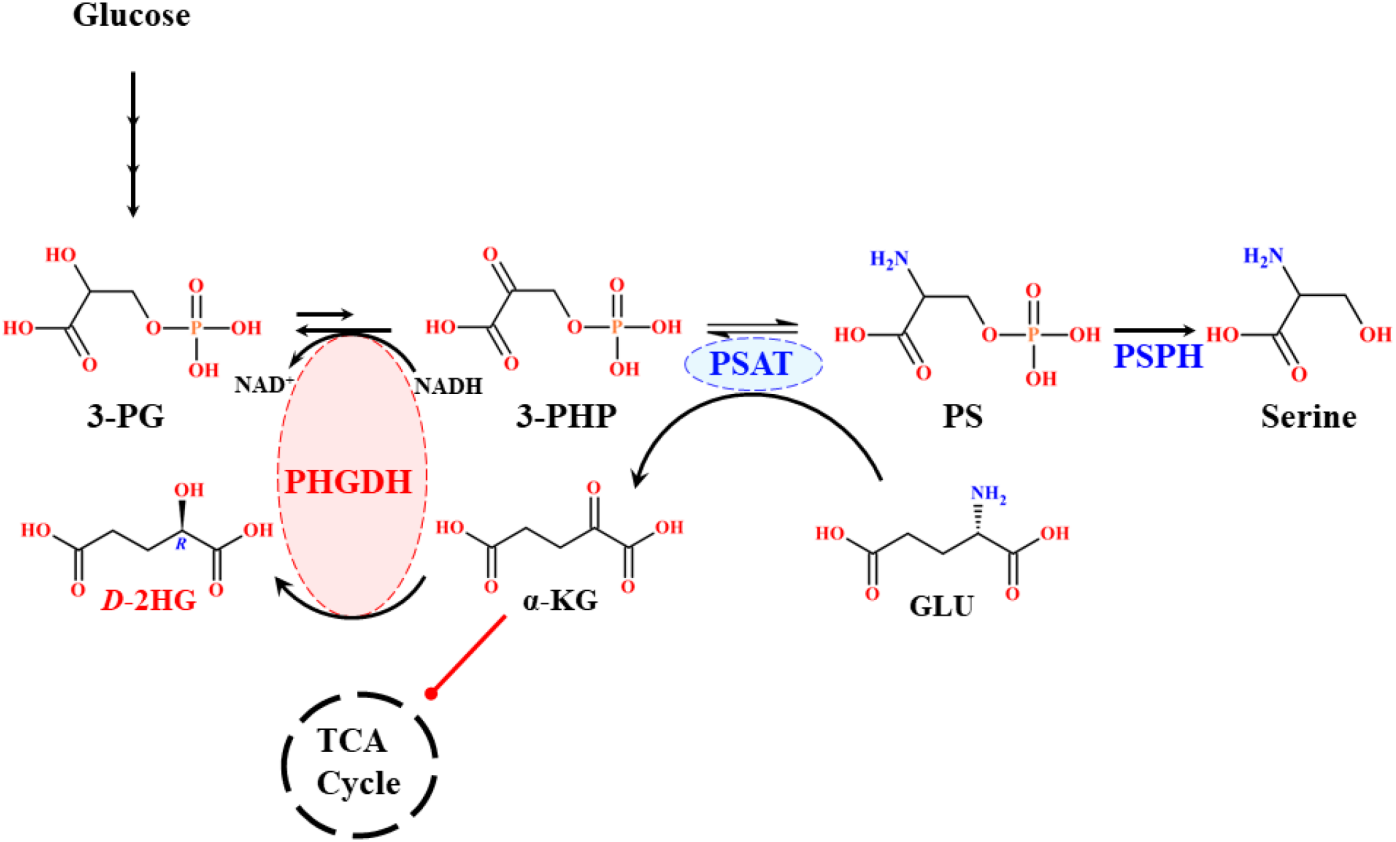
Mechanistic route of the overexpressed PHGDH reaction pathway.

PHGDH impairment has broad metabolic consequences, affecting serine availability, the TCA cycle anaplerosis, redox balance, energy metabolism, nutrient sensing, and cell signaling (59). Mechanistically, SSP intermediates couple to the TCA cycle, where PSAT1 catalyzes the conversion of 3-PHP to PS while simultaneously converting GLU to α-KG, thereby channeling GLU-derived carbon into the TCA flux (58). This reaction is nitrogen-neutral and avoids ammonia release, unlike the glutamate dehydrogenase pathway, which generates toxic ammonia (59). Approximately 50% of net GLU to α-KG conversion occurs via PHGDH-PSAT, highlighting PHGDH’s role not only in serine biosynthesis but also in broader metabolic regulation (60). Overexpression of PHGDH is frequently observed in cancers and represents an emerging therapeutic target. Elevated PHGDH has been reported in squamous cell carcinoma, colon carcinoma, sarcoma, cervical, glioma, glioblastoma, astrocytoma, gastric, pancreatic, liver, kidneys, lungs, ovarian, and highly expressed in breast and skin cancers (57, 61, 62). Its upregulation effectively diverts glycolytic flux toward biomass production, supporting tumor growth and oncogene activation (57). Clinical studies report PHGDH overexpression in 16% of all cancers, 20-25% of breast cancers, 40% of melanomas, 68% of estrogen receptor (ER)-positive breast tumors, and up to 70% of ER-negative and triple-negative breast cancers (58). Alarmingly, PHGDH overexpression in 50% of breast cancers reduces patient survival to <5 years despite treatment (63). Functionally, PHGDH belongs to the 2-hydroxyacid dehydrogenase family and can generate stereospecific *D*-isomer metabolites. Remarkably, 3-PHP (its canonical substrate) and GLU-derived α-KG share structural similarities, enabling overexpressed PHGDH to aberrantly reduce α-KG (using NADH) to *D*-2HG and NAD^+^, rather than processing 3-PHP to 3-PG. This stereospecific misreaction significantly increases *D*-2HG level in cells (**Schemes 3**, **4**).

Binding studies indicate that α-KG has nearly 3-fold higher affinity than 3-PHP/3-PG (64). Consequently, amplified PHGDH strongly enhances *D*-2HG formation through a thermodynamically favored reverse reaction, with profound consequences for cell signaling, metabolism, and epigenetic regulation. Experimental evidence supports these findings, where PHGDH knockdown reduces cellular *D*-2HG levels by 50% in PHGDH-overexpressing breast cancers, while overexpression increases *D*-2HG levels by 2-fold compared to wild-type PHGDH (64). In breast and pancreatic cancer cells, PHGDH overexpression drives serine/glycine *de novo* biosynthesis, whereas knockdown significantly impairs proliferation, even in the presence of extracellular serine. Moreover, PHGDH upregulation correlates with increased ROS generation, oxidative stress, and hypoxia-inducible factor-1α (HIF-1α) activation, all of which promote metastasis in animal and cellular models (56, 57). Redirecting glycolytic flux creates metabolic abnormalities and increases PHGDH activity. Genomic amplification of PHGDH has also been linked to the transcriptional regulation of tumor suppressor genes and post-translational modifications via proteasomal degradation, thereby further promoting oncogenic pathways (57, 61). Overall, PHGDH acts as both a driver of serine biosynthesis and a noncanonical source of *D*-2HG, linking glycolysis, serine metabolism, and oncometabolite production. While its role in biomass generation is well established, the precise mechanisms underlying PHGDH-mediated *D*-2HG elevation and its impact on cancer metabolism remain incompletely understood, warranting further investigation.

### 2.2 Hypoxia-mediated 2HG

Various reaction conditions, including solvent, temperature, pH, oxygen concentration, and availability of substrate or cofactor, can profoundly influence enzyme function. These factors modulate the conformational flexibility of enzyme active sites, altering their catalytic efficiency. Fluctuations in pH and oxygen levels within the cellular compartment significantly affect enzyme kinetics, often enhancing enzyme promiscuity. Such alterations promote abnormal biochemical reactions that contribute to pathogenic mechanisms, whereas other stressors may denature or inactivate enzymes (65). In clinical terms, cellular hypoxia is defined as the deprivation of adequate oxygen. Hypoxia is a critical determinant of biochemical processes across species, contributing to the development and progression of multiple diseases, including atherosclerosis, ischemic heart disease, chronic lung disease, blood-brain barrier dysfunction leading to stroke, central nervous system (CNS) impairment, oncogene activation, and cancer cell proliferation (66). Collectively, hypoxia is one of the major contributors to global mortality. Beyond its pathological role, hypoxia also serves as an essential physiological regulator of development and tissue repair. However, under sustained or severe conditions, it rewires cellular metabolism, disrupting enzyme activity involved in energy production and redox homeostasis. This impacts glycolysis, TCA cycle intermediates, precursor biosynthesis, tumor suppressor enzyme activity, and the ETC, while simultaneously increasing ROS production, tumor proliferation, and oncogene activation (66). Hypoxia is particularly relevant in cancer, where more than 60% of solid tumors exhibit oxygen partial pressures below 1% (67). Insufficient perfusion of blood and oxygen diffusion within the tumor microenvironment further exacerbate hypoxia, thereby promoting tumor expansion. A key molecular mediator of hypoxia is the hypoxia-inducible factor (HIF) family of transcription factors. Hypoxia stabilizes HIF, which orchestrates transcriptional programs that drive angiogenesis, metabolic modification, invasion, and metastasis, thereby increasing tumor lethality. In humans, there are three HIF family members (HIF-1, HIF-2, and HIF-3), each composed of an oxygen-sensitive α-subunit and an oxygen-independent β-subunit, which form heterodimeric DNA-binding complexes. These complexes regulate gene transcription to support adaptation to low oxygen levels, influencing processes such as migration, vasodilation, cell signaling, and survival. Of these, HIF-1α is the most widely studied hub that controls many transcriptional factors and molecular signaling pathways to enhance tumorigenesis and is considered a central regulator of oncogene activation (68).

**Figure 1:**
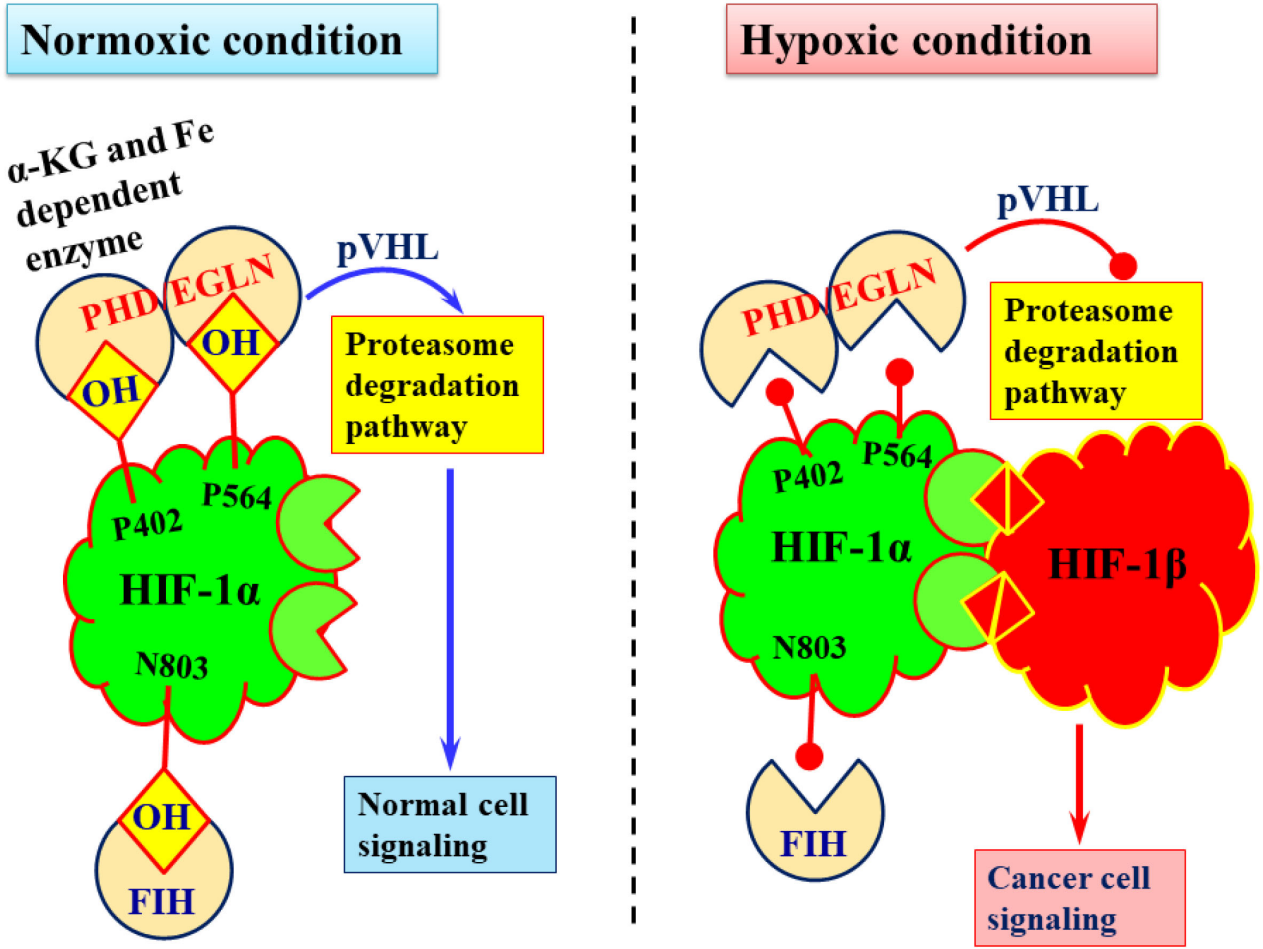
Pictorial representation of HIF-1α stabilization for cancers.

In normoxic conditions, HIF-1α is unstable and rapidly degraded. This occurs through hydroxylation of three critical residues: two proline residues within the oxygen-dependent degradation domain (Pro402 and Pro564), hydroxylated by HIF prolyl hydroxylases (PHD/EGLN), and one asparagine residue (Asn803) within the C-terminal transactivation domain, hydroxylated by factor inhibiting HIF (FIH). PHD/EGLN are non-heme, iron and α-KG-dependent dioxygenases that hydroxylate HIF-1α, promoting its recognition by the von Hippel-Lindau (VHL) tumor suppressor protein and subsequent proteasomal degradation (**Figure 1**). Meanwhile, FIH hydroxylates Asn803, preventing interaction with the transcriptional co-activator p300, thereby destabilizing HIF-1α (68, 69). Under hypoxic conditions, hydroxylation by PHDs and FIH is suppressed, preventing proteasomal degradation. Stabilized HIF-1α translocates to the nucleus, where it forms a heterodimeric transcriptional complex with HIF-1β. This complex binds to hypoxia response elements (HREs) and activates the expression of 100-200 genes that drive pathogenic and oncogenic pathways (68). The stability of HIF-1α can also be enhanced by additional factors such as PHD inactivation, FIH suppression, iron deficiency, α-KG depletion, mutations in VHL, and the aggregation of oncometabolites like 2HG. Nonetheless, hypoxia remains the principal driver of HIF-1α stabilization and subsequent reprogramming of cellular metabolism. Most cancer cells and metastatic tumors are strongly associated with glycolytic rewiring and TCA cycle impairment (56, 66, 69). Therefore, in this section, we focus on hypoxia-associated alterations in cellular metabolism to promote 2HG and cancer progression.

From the perspective of energy metabolism, glucose and TCA intermediates are indispensable substrates that participate in numerous cellular processes, including glycogenesis, glycogenolysis, gluconeogenesis, glycoconjugate biosynthesis, amino acid production, fatty acid synthesis, DNA repair, protein and lipid biosynthesis, and epigenetic modification. Glycolysis is the central catabolic pathway of glucose, producing ATP and generating acetyl-CoA, which serves as the primary carbon source for the TCA cycle. The TCA cycle, in turn, supports mitochondrial respiration, oxidative phosphorylation, and electron transport, while maintaining redox signaling and biosynthetic pathways essential for cell survival (23). HIF-1α signaling plays a pivotal role in diverting glucose metabolism toward LA fermentation rather than oxidative phosphorylation. This shift is achieved by the upregulation of multiple glycolytic enzymes and transporters, including glucose transporters (GLUT1, GLUT3), lactate dehydrogenase (LDH), malate dehydrogenase (MDH1, MDH2), PKM2, pyruvate dehydrogenase kinase (PDK), hexokinases (HK1, HK2), phosphoglycerate kinase 1 (PGK1), and aldolases A and C (ALD-A, ALD-C) (70, 71). Among these, LDHA and MDH1/2 are of particular importance, as they directly catalyze reactions that lead to the formation of *L*-2HG, but not the *D*-enantiomer, under hypoxic conditions (71). Thus, hypoxia-induced metabolic rewiring contributes significantly to oncometabolite sustainability, with enantioselective consequences for cancer progression.

#### 2.2.1 Lactate dehydrogenase A

**Scheme 5:**
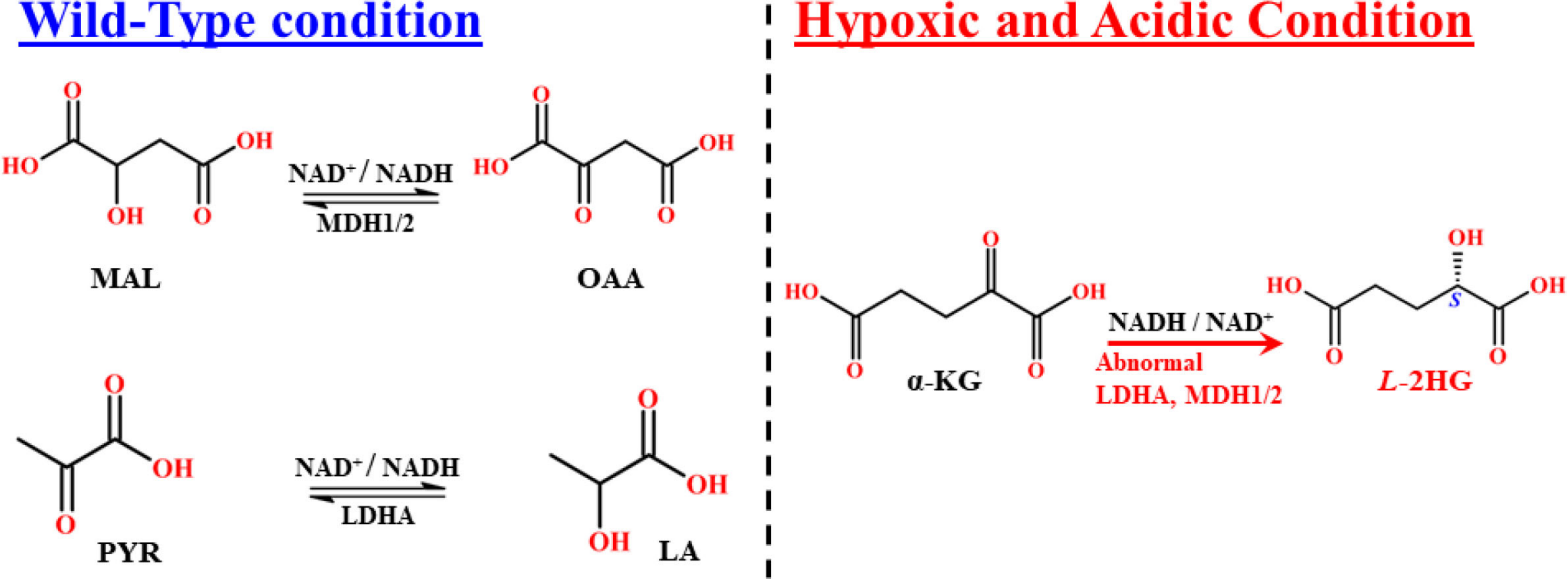
Mechanistic route of the wild-type and acidic, hypoxic-mediated MDH1/2, and LDHA reaction pathway.

The terminal step of glycolysis is catalyzed by LDH, an enzyme strongly associated with metabolic acidosis and HIF-1α signaling. LDH catalyzes the reversible interconversion of pyruvate (PYR) and LA, coupled with the oxidation of NADH to NAD^+^. Kinetic studies indicate that LDH follows an ordered mechanism, in which NAD^+^/NADH binding precedes substrate interaction, similar to MDH (72). LDH is a ubiquitous oxidoreductase present in nearly all tissues, with high expression in muscle, liver, and kidney. It functions primarily in the cytosol to regulate PYR levels, thereby influencing gluconeogenesis and nucleotide metabolism. Structurally, LDH exists as a tetrameric enzyme with five isoforms (LDH-1 to LDH-5), composed of different combinations of four subunits: LDHA, LDHB, LDHC, and LDHD. The two principal functional subunits are muscle (M) and heart (H). Although all isoforms catalyze the same reaction, they differ in substrate affinity, regulation, and tissue distribution. During intense muscular activity, LDH supports anaerobic glycolysis through the Cori cycle. Elevated LDH activity is a pathogenic hallmark, reported in multiple conditions including liver, heart, blood, bone, muscle, and brain disorders, as well as spinal cord inflammation, HIV infection, and numerous cancers (73). Among isoforms, LDHA is particularly important in cancer, where it is consistently upregulated under hypoxia and altered pH. Our focus here is to critically examine the role of LDHA in hypoxia and acidic conditions, in 2HG formation at the cytosolic compartment. The LDHA gene, located at 11p15.1, encodes the *L-*isomerase that converts PYR to *L-*lactate. LDHA is composed of 332 amino acids with a molecular mass of 36.689 kDa. In glycolysis, PYR is the final cytosolic product of glucose metabolism and serves as a key intermediate, undergoing oxidative decarboxylation by the PDH complex to generate acetyl-CoA. Acetyl-CoA then enters the mitochondrial pool, where it serves as a significant carbon source for the TCA cycle (73, 74). Blockage of acetyl-CoA entry into the TCA cycle impairs survival by increasing ROS production (via ETC dysfunction) and enhancing enzymatic deregulation, ultimately promoting tumor cell activation. In normoxia, tumor cells utilize oxygen for differentiation and proliferation, with PDH activity channeling PYR into acetyl-CoA. However, under hypoxia, PDH is inhibited by PDK, diverting PYR toward LDHA-mediated conversion into LA. This creates a pseudo-hypoxic environment that supports tumor survival by sustaining energy and nutrient supply. LDHA, a direct HIF-1α-encoded enzyme, is upregulated during hypoxia to facilitate this metabolic switch. By inhibiting PYR entry into the TCA cycle and favoring LA production, LDHA disturbs mitochondrial respiration and promotes ROS intensification, thereby fueling cancer proliferation (74). Beyond its canonical role, LDHA is upregulated and acquires non-specific reductive activity under HIF-1α signaling. In this context, LDHA shifts substrate specificity from PYR to α-KG, reducing α-KG to *L*-2HG in the presence of excess α-KG (71) (**Scheme 5**).

Clinically, α-KG is recognized as a key precursor of *L*-2HG. Indeed, intracellular α-KG and its reduced metabolite *L*-2HG increase by up to 2-25 fold under hypoxic conditions (71). Thus, hypoxia-driven LDHA activity is a fervent source of *L*-2HG in cancer cells. The cellular redox environment plays a central role in this process. Biochemical reactions rely heavily on electron transfer mediated by NAD^+^/NADH and NADP^+^/NADPH couples, which not only serve as cofactors but also act as substrates in enzymatic reactions, sustaining redox homeostasis and cellular energetics. For example, NAD^+^ acts as an essential electron sink to mediate the glycolysis process, and NADH acts as an electron carrier to catalyze the oxidative phosphorylation reaction in the mitochondrial compartment. Similarly, NADP^+^ and NADPH support cytoplasmic biosynthesis of lipids, fatty acids, and nucleic acids. Redox couples also detoxify ROS species, including peroxides, superoxides, hydroxyl radicals, and singlet oxygen. Metabolic imbalance of redox couple is highly connected with the various pathogenic processes, including cardiovascular disease, aging, neurodegenerative disease, and cancers (75). The redox balance is usually maintained and transferred through transmembrane shuttles to the respective compartment. One of the most important is the α-KG solute carrier protein *SLC*25A11 (also known as the malate/α-KG carrier), which exchanges malate (MAL) and α-KG between cytosol and mitochondria, facilitating NAD^+^/NADH transfer and supporting glycolysis and oxidative phosphorylation (76).

In mitochondria, α-KG is produced mainly via GLU anaplerosis. Excess GLU-derived α-KG is converted to ICT by IDH2, with concomitant oxidation of NADPH to NADP^+^. This α-KG and ICT interconversion, along with the NADPH/NADP^+^ and NADH/NAD^+^ ratios, is tightly regulated by nicotinamide nucleotide transhydrogenase (NNT). NNT catalyzes hydride transfer from NADH to NADP^+^, generating NADPH and NAD^+^, thereby supporting mitochondrial redox balance and ETC function. NNT contributes up to 45% of mitochondrial NADPH, making it crucial for ROS suppression and mitochondrial stability. In hypoxia, the stabilized HIF-1α disrupts NNT activity, reducing NADPH availability while increasing mitochondrial NADH. This imbalance impairs the α-KG to ICT conversion, deregulating the TCA cycle. Elevated NADH exacerbates reductive stress, enhances ROS formation, and drives TCA dysfunction (77). The unreacted GLU-derived α-KG is shuttled to the cytosol via *SLC*25A11, conserving metabolic equilibrium. In the cytosol, HIF-1α signaling upregulates LDHA, thereby increasing NADH availability. This complete combination promotes LDHA-mediated reduction of GLU-derived α-KG to *L*-2HG, producing NAD^+^ as a byproduct and disrupting normal cellular processes (71). Metabolic and isotopic tracing studies confirm that endogenous *L*-2HG produced under hypoxia via LDHA originates predominantly from GLU-derived α-KG. However, the precise regulation of excess α-KG availability in the cytosol remains a topic of debate. Regardless, the reproduction of *L*-2HG in the cytosolic compartment is difficult to eliminate, and its persistence can lead to the aggressive reprogramming of metabolism to fuel uncontrolled cancer cell proliferation.

#### 2.2.2 Malate dehydrogenase (MDH1/2)

Malate (MAL) is a central metabolite that participates in numerous biochemical reactions to sustain cellular function and redox balance. Its intracellular concentration is tightly regulated by cytosolic and mitochondrial MDH enzymes, which play critical roles in TCA cycle regulation, redox homeostasis, and cell signaling. MDH is a ubiquitous NADH dependent *L*-enantiomeric enzyme that catalyzes the reversible reduction of oxaloacetate(OAA) to *L*-MAL, accompanied by the oxidation of NADH to NAD^+^, thereby maintaining the NAD^+^/NADH ratio (78). *In vitro* studies have shown that MDH kinetically and thermodynamically favors the reduction of OAA to *L*-MAL rather than the reverse reaction (79). MDH is stereospecific, accepting only the *L*-MAL as a substrate, while *D*-MAL acts as an inhibitor (78). Two isoforms of MDH exist in eukaryotes, where MDH1, located in the cytosol, and MDH2, localized to mitochondria. Both catalyze the same reaction to sustain MAL and redox pools in their respective compartments. MDH enzymes are abundantly expressed in the brain, skeletal muscle, and heart, with lower expression in the liver, kidneys, and smooth muscle (80). MDH1 and MDH2 share similar catalytic functions and kinetics, with high structural homology and homodimeric organization. Each subunit consists of two domains, an N-terminal NAD-binding domain and a C-terminal substrate-binding domain. The active site forms at the interface of these domains, stabilized by key amino acid residues (81). Both isoforms are synthesized in the cytoplasm. MDH1 remains unchanged after N-terminal acetylation, while MDH2 undergoes N-terminal extension and subsequent import into mitochondria (82). Structurally, MDH1 (334 amino acids, 36.426 kDa; gene locus 2p15) is more polar and acidic than MDH2 (338 amino acids, 35.503 kDa; gene locus 7q11) due to higher numbers of charged residues (78, 83). Interestingly, mitochondrial MDH2 can dissociate into inactive monomers at acidic pH (≤5), whereas MDH1 remains dimeric and active, suggesting that MDH1 is more resilient under acidic conditions, making it more relevant in pathological and cancerous states (84). The catalytic mechanism of MDH involves proton transfer mediated by Asp152 and His168, which form a proton relay system between solvent and substrate. This pair also enhances hydride transfer from NADH to OAA. Notably, the same catalytic pair is conserved in LDHA, highlighting mechanistic similarities (85). Indeed, MDH1 and LDHA share high sequence similarity, and even a single amino acid change, Arg100 in LDHA versus Gln100 in MDH1, can potentially alter substrate specificity. In conditions where LDHA is impaired or absent, MDH1 can act as a surrogate, catalyzing PYR to LA conversion instead of its canonical OAA to MAL reaction (81).

A central role of MDH enzymes lies in the malate-aspartate shuttle (MAS), which is crucial for the function of the TCA cycle, maintaining redox balance, gluconeogenesis, amino acid synthesis, and metabolite exchange between cytosol and mitochondria. The MAS maintains NAD^+^/NADH homeostasis across compartments, which is essential for both glycolysis and mitochondrial respiration. Mechanistically, MDH1 regenerates NAD^+^ in the cytosol by reducing OAA to MAL, supporting glycolysis through glyceraldehyde-3-phosphate dehydrogenase. MDH2 catalyzes the reverse reaction, oxidizing MAL to OAA and generating NADH, which fuels oxidative phosphorylation. Together, MDH1/2 maintain compartment-specific NAD^+^/NADH pools, with metabolite exchange (*L*-MAL and OAA) facilitated by four key proteins, glutamic-oxaloacetic transaminases (GOT1 and GOT2), citrate carrier proteins *SLC*25A11, and aspartate-glutamate carrier proteins (*SLC*25A12 or *SLC*25A13). GOT1 catalyzes cytosolic aspartate to OAA conversion with concomitant transamination of α-KG to GLU, while GOT2 mediates the reverse in mitochondria. Carrier proteins *SLC*25A12 or *SLC*25A13 transport aspartate and GLU across membranes, while MDH1 converts excess cytosolic OAA to MAL, and mitochondrial GOT2 mediates α-KG, which is potentially relocated with the help of mitochondrial membrane protein *SLC*25A11 to maintain the metabolites equilibrium (83).

Impairment of the MAS disrupts metabolite balance, leading to the overproduction of ROS and promoting disease states. In cancer, MDH1 deregulation has been linked to autophagy-associated energy transfer and tumor proliferation (83). Under hypoxic conditions, stabilized HIF-1α upregulates MDH expression, which, in turn, alters enzyme activity to promote the side reaction of α-KG to *L*-2HG. Although this occurs at a catalytic efficiency 10^7^-10^8^ times lower than the primary reaction (86) (**Scheme 5**), even a minimal *L*-2HG level can stimulate oncogenic pathways. Elevated MDH activity under hypoxia has been associated with the lungs (87), renal (88), and pancreatic cancers (89), as well as osteoclastogenesis (90), neurodevelopmental delay, senescence, and other pathologies (80, 91). The oncogenic impact of MDH2 is amplified by its suppression of the repair enzyme L-2-hydroxyglutarate dehydrogenase (L2HGDH), whose activity is reduced by up to 50% under hypoxic conditions, particularly in mitochondria. This inhibition exacerbates TCA cycle dysfunction, mitochondrial impairment, and ROS enhancement (92). Metabolic tracing studies confirm that GLU-derived α-KG is the predominant precursor for *L*-2HG formation in hypoxia (93). Overexpression of MDH2 under these conditions increases OAA, which can be transaminated to aspartate, while GLU derived α-KG is simultaneously diverted to *L*-2HG formation (94). Excess α-KG also translocates into the cytoplasm via the carrier protein *SLC*25A11, where cytosolic MDH1 further converts it into *L*-2HG, in concert with GOT1 (71, 83). The combined effects of elevated MDH activity, excess α-KG, hypoxia, and reduced L2HGDH repair capacity result in persistent viability of *L*-2HG. This oncometabolite, in turn, drives oncogene activation and malignant progression across multiple cancer types.

### 2.3 Metabolic acidosis

The role of hypoxia in cancer is not limited to enzymatic upregulation but is intimately linked to metabolic acidosis. Acidic pH changes in the tumor microenvironment, often referred to as metabolic acidosis, are considered the “twin sister” of hypoxia. Indeed, hypoxia commonly drives acidosis, and acidosis can in turn intensify hypoxia, creating a vicious cycle. This interplay is mediated through HIF-1α stabilization and the upregulation of Sodium-Hydrogen Exchangers (Na^+^/H^+^ or NHEs), Monocarboxylate Transporter 4 (MCT4), and Carbonic Anhydrase IX (CAIX) (95). Thus, hypoxia fosters acidosis, and acidosis in turn reinforces hypoxia (21). Regulation of pH is therefore strongly connected to HIF-1α stabilization and its downstream effectors. Under hypoxic or acidic conditions, glycolysis is upregulated (Warburg effect), driving LDHA and MDH overexpression. This results in overproduction of LA, protons (H^+^), and oxidative equivalents (NADH), hallmarks of metabolic acidosis (96). In acidic environments, excess free protons alter buffering capacity and promote metabolic protonation (97). The α-KG is a weak acid that undergoes protonation in acidic conditions (pH ≤ 6). Protonated α-KG has an increased tendency to interact with the LDHA substrate-binding pocket, particularly at residue Gln100, through strong hydrogen bonding (98). This unusual interaction shifts LDHA activity away from its canonical PYR to LA reaction toward the reduction of GLU-derived α-KG into *L*-2HG. Similarly, low pH destabilizes mitochondrial MDH2 dimers into inactive monomers, while cytosolic MDH1 remains stable and catalytically active toward α-KG reduction, further promoting *L*-2HG formation (84). Although MDH1-mediated *L*-2HG production is modest compared to LDHA, it becomes more significant under acidic stress.

*In vitro* studies demonstrate that even under normoxic conditions, low pH enables LDHA to catalyze the conversion of α-KG into *L*-2HG at levels up to 4-fold higher, supporting HIF-1α stabilization in the absence of oxygen (98). Conversely, LDHA deficiency markedly reduces *L*-2HG production and destabilizes HIF-1α and its downstream pathways. Thus, metabolic acidosis promotes HIF-1α stabilization via LDHA-mediated *L*-2HG formation. This plays a crucial role in immune cell survival and the activation of pathogenic processes. Medical literature reports that pancreatic, stromal, and renal tumors induced by hypoxia exhibit strong LDHA upregulation, with endogenous *L*-2HG serving as a potential biomarker for these cancers. Elevated *L*-2HG amplifies oxidative stress, alters reductive equivalents, and drives ROS production. It also disrupts antioxidant defenses, induces endoplasmic reticulum (EPR) stress, and promotes cellular dysfunction, leading to tissue impairment, neuronal damage, inborn errors of metabolism, and the amplification of toxic metabolites that generate free radicals (71). The highly reactive free radicals produced in acidosis oxidize proteins, lipids, and cell membranes, accelerating metabolic deregulation and triggering cell death. DNA and RNA are also damaged via purine and pyrimidine oxidation, protein-DNA crosslinks, single- and double-strand breaks, and RNA folding perturbations, all of which compromise gene function and signal transduction (99). Both enantiomers of 2HG act as potent inhibitors of α-KG-dependent dioxygenases, reprogramming metabolic processes and promoting oncogenic pathways that limit survival. Notably, *L*-2HG is at least 5-10 fold more potent than *D*-2HG in inhibiting α-KG-dependent enzymes, making it a stronger oncogenic driver (21). For example, high levels of *L*-2HG in pancreatic and stromal cancers promote self-renewal properties, thereby accelerating therapeutic resistance (100). Furthermore, urinary metabolomics studies have identified elevated *L*-2HG as a key biomarker for electron transport defects (ETDs), particularly in children. Importantly, these ETD-related disorders arise from *L*-2HG, not *D*-2HG *(*92).

### 2.4 The role of repair and rewiring mechanisms

In biochemical processes, deviations from wild-type reactions occasionally occur, resulting in the production of aberrant or error products at low concentrations. Such disturbances may arise from changes in temperature, metabolic acidosis, hypoxia, pH fluctuations, alterations in the glycolytic pathway, changes in substrate or cofactor availability, and enzyme instability or mutation. Among these factors, enzyme mutations are the primary drivers of aberrant product formation, while the other conditions often act indirectly by altering enzyme conformation and promoting deviant catalytic routes. Enzyme instability (“tautness”) is particularly critical, as it can disrupt entire biological systems by redirecting reactions toward unfavorable mechanistic pathways that stimulate disease progression. These conformational changes can also affect effector or activator binding, alter substrate affinity, or compromise enzyme concentration, ultimately leading to denaturation, mutations, or alternative side reactions. As a result, aberrant products may form, acting as inhibitors by binding to the active sites of other enzymes, thereby disrupting downstream biotransformation processes and enriching pathogenic pathways. For example, the oncometabolite 2HG, when formed under wild-type conditions due to changes in pH or hypoxia, can interfere with tumor suppressor proteins such as TP53. This interaction fosters uncontrolled tumor proliferation and has been reported across various cancers. Although enzymes are inherently prone to error under fluctuating biological conditions, cells possess repair and rewiring mechanisms that safeguard against such errors. These systems regulate the cellular milieu, maintain wild-type processes, and restore balance by removing unwanted metabolites, thereby reducing toxicity and preventing the formation of deviant products. Importantly, several repair pathways specifically counteract toxic oncometabolites, such as 2HG. Within this context, two key mitochondrial enzymes, D2HGDH and L2HGDH, play central roles in detoxification. These enzymes catalyze the reconversion of aberrant *D*-2HG and *L*-2HG back to α-KG, thereby restoring metabolic balance and protecting against oncogenic progression (21, 71, 101). The following sections will examine each enzyme in detail, beginning with D2HGDH and then L2HGDH.

#### 2.4.1 D-2-hydroxyglutarate dehydrogenase

**Scheme 6:**
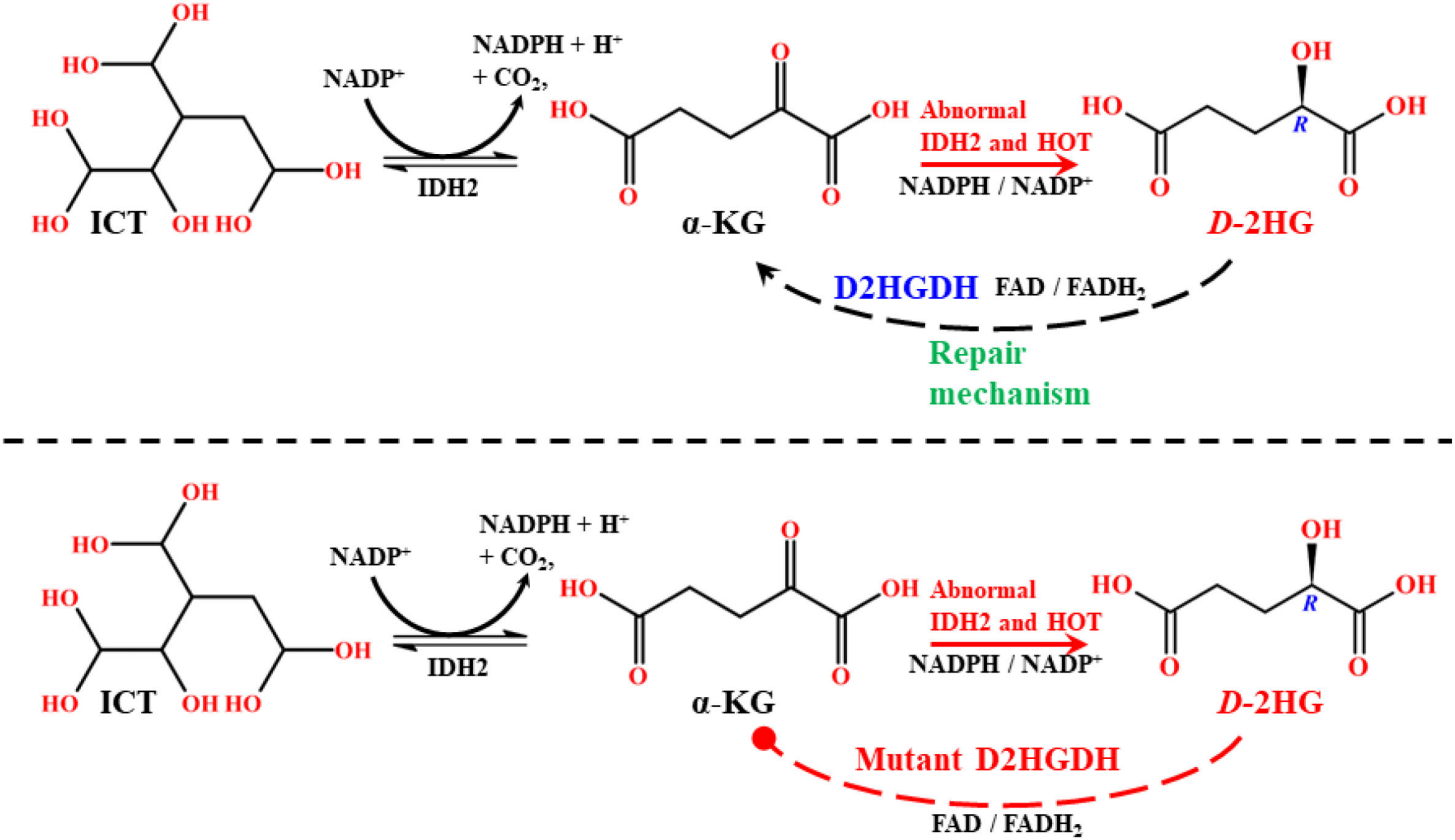
Mechanistic route of the wild-type and mutant type D2HGDH reaction pathway.

The defensive enzyme D2HGDH is a mitochondrial flavin adenine dinucleotide (FAD)-dependent enzyme that plays a pivotal role in purifying *D*-2HG. It consists of 521 amino acids, has a molecular mass of 56.416 kDa, and is encoded on chromosome 2q37.3. Its primary function is to reduce *D*-2HG level by oxidizing it back to α-KG, thereby restoring cellular homeostasis and supporting TCA cycle activity. The repair mechanism involves electron transfer via the FAD cofactor within the mitochondrial matrix (102) (**Scheme 6**). D2HGDH is expressed at high levels in the liver and kidney, with moderate expression in the heart and brain (103). Its central role is to contribute to metabolic pathways and energy maintenance by regenerating α-KG for the TCA cycle. Disruption of D2HGDH profoundly impacts cellular metabolism, leading to disorders that include several cancer types (102). Like many enzymes, the activity of D2HGDH is susceptible to perturbation by pH, temperature, and cellular environment (21). However, mutations in D2HGDH are particularly detrimental, as they abolish its repair function, resulting in pathological construction of *D*-2HG *(*53). A key mechanistic connection exists between D2HGDH and IDH2, where mutations in IDH2 generate *D*-2HG from α-KG, while D2HGDH normally converts this excess *D*-2HG back into α-KG. Mutations or inactivation of D2HGDH severely impair this repair loop, resulting in persistent *D*-2HG. In fact, *D*-2HG concentrations are 2-8 fold higher in IDH2 mutant tumors compared to conditions where D2HGDH is merely downregulated (53). More than 30 distinct mutations have been identified in the D2HGDH gene, including homozygous, heterozygous, and frameshift variants, all of which led to elevated *D*-2HG levels. Among these, germline missense mutations I147S and V444A are especially significant. These variants suppress catalytic activity by disrupting the FAD binding site, producing an apo-protein form that is catalytically inactive (104). The inactive enzyme fails to oxidize *D*-2HG, resulting in the unchecked availability within the mitochondrial and cellular compartments. Experimental studies show that knockdown of these variants restores α-KG levels and tumor suppressor activity, thereby re-establishing wild-type cellular functions through *D*-2HG depletion (105). Thus, while D2HGDH typically serves as a prophylactic against aberrant metabolite expansion, its disruption represents a critical vulnerability that promotes the development of cancer. The systematic study of D2HGDH mutations and their role in modulating *D*-2HG levels remains an important area of ongoing research (106).

#### 2.4.2 L-2-hydroxyglutarate dehydrogenase

**Scheme 7:**
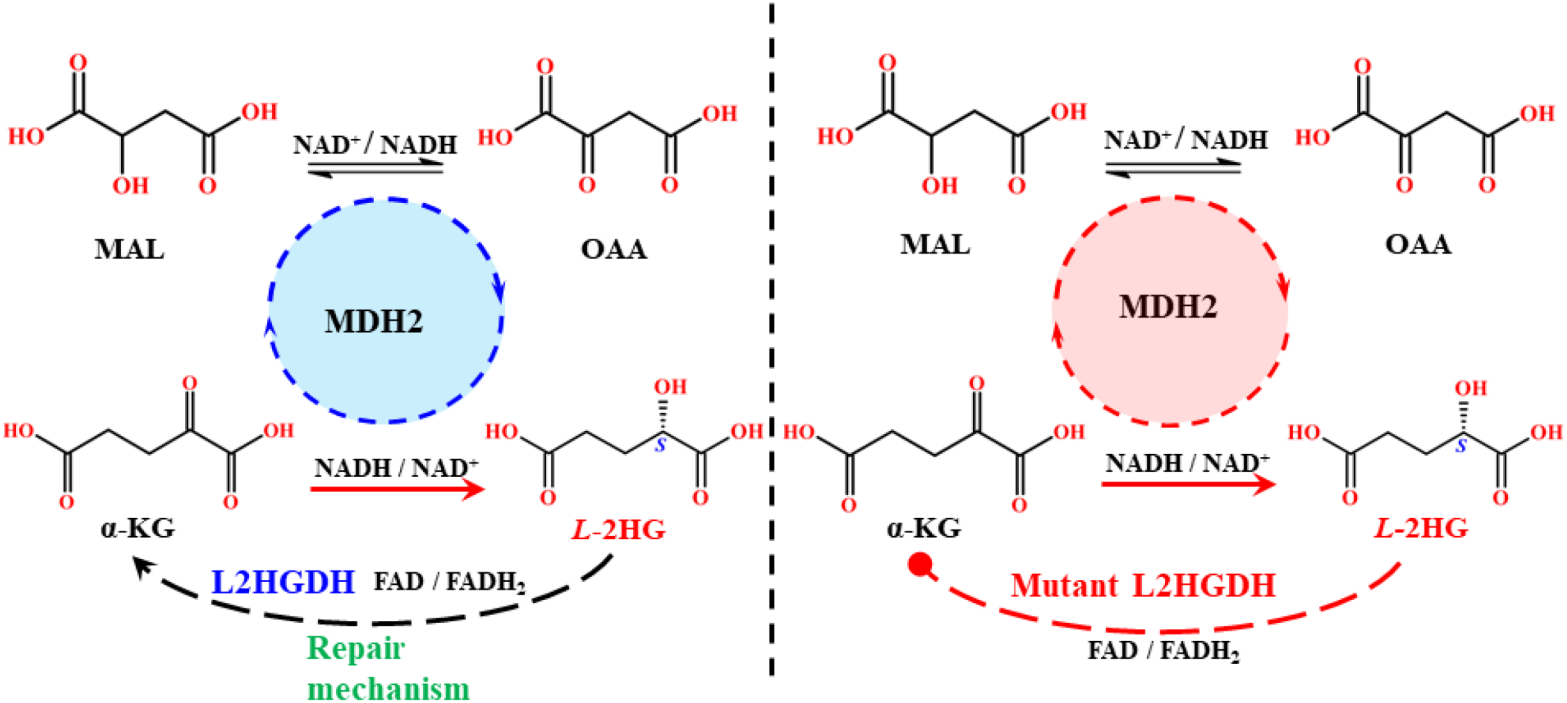
Mechanistic route of the wild-type and mutant type L2HGDH reaction pathway.

The protective enzyme L2HGDH, like D2HGDH, is a mitochondrial FAD-dependent protein that shields against oncometabolite enrichment. L2HGDH also shows NAD^+^/NADP^+^ dependence, particularly in liver mitochondria. Its fundamental role is to lower the cellular concentration of *L*-2HG by oxidizing it back into α-KG through a repair mechanism (**Scheme 7**). This cleansing activity is prominent in the brain, heart, liver, kidney, testis, and muscle (86).

L2HGDH consists of 521 amino acids, has a molecular mass of 56.416 kDa, and is encoded on chromosome 14q22.1 (107). Loss of function in this enzyme significantly compromises regular cellular activity, impairs repair capacity, and causes elevated *L*-2HG levels, thereby strongly linking it to tumorigenesis and other pathogenic pathways. Enzyme deregulation may occur due to mutation, hypoxia, pH changes, ROS elevation, antioxidant imbalance, temperature fluctuations, or general alterations in the cellular milieu. Clinical evidence shows that mitochondrial L2HGDH is frequently affected by homozygous germline mutations, leading to markedly elevated *L*-2HG levels in body fluids (94, 108). More than 53 mutations have been identified, with several recurring variants significantly impairing enzyme kinetics. Key mutations include L18R, G55N, G57R, G60R, H98R, K136R, and P302L, all of which are associated with overexpression of *L*-2HG *(*107, 109–114). Among these, K136R and G60R are particularly important, as they directly disrupt the FAD/NAD(P) cofactor-binding pocket, resulting in complete enzyme inactivation (107, 112).

Loss of L2HGDH activity impacts a broad range of cellular processes, including energy maintenance, electron transport, and redox balance. Its impairment is associated with multiple diseases, particularly cancers such as lung cancer, colorectal cancer, nasopharyngeal carcinoma, clear cell renal cell carcinoma, and brain tumors (21, 94). Importantly, L2HGDH deficiency strongly predisposes to brain malignancies, including glioma, glioblastoma, medulloblastoma, low-grade astrocytoma, Wilms tumor, primitive neuroectodermal tumors (PNETs), and thalamic tumors. These conditions are frequently reported in children and infants, underscoring the pathological significance of *L*-2HG extension in early life (115). Elevated *L*-2HG has also been linked to pregnancy termination and complications in childbirth (116). Redox imbalance further aggravates disease progression. Impairment of L2HGDH consumes large amounts of NADH/NAD^+^, leading to a disturbance of the NADH/NAD^+^ availability, particularly in the liver. This imbalance induces reductive stress, driving mitochondrial dysfunction and impairing hepatic activity (117). Compared to *D*-2HG, *L*-2HG has a more pronounced impact on brain function, making it a highly pathogenic metabolite (115). Indeed, *L*-2HG is considered more potent than *D*-2HG, with both enantiomers recognized as antagonists of mammalian cellular function and potent oncometabolites. Clinical evidence also highlights the link between L2HGDH impairment and antioxidant systems. Loss of function of this enzyme impairs primary antioxidants, including superoxide dismutase, glutathione peroxidase, and glutathione, leading to reduced antioxidant defenses, increased ROS levels, and oxidative stress. Excessive ROS generation, including superoxide, peroxides, and free radicals, disrupts cellular function, promotes cell death, and contributes to neurodegenerative diseases such as Alzheimer’s disease, Parkinson’s disease, and epilepsy (21, 118).

At the mechanistic level, both 2HG enantiomers act as competitive inhibitors of α-KG-dependent dioxygenases, but *L*-2HG is particularly potent. Acting as a structural analog of GLU, *L*-2HG inhibits diverse biochemical reactions and interferes with neurotransmitter processes (119). This may explain its pronounced neurological effects compared to *D*-2HG. Overall, while the cellular pathways of *L*-2HG metabolism remain incompletely understood, the impairment of L2HGDH clearly plays a central role in disease progression. Given its broad pathological impact, L2HGDH and its associated pathways remain a therapeutic target of great interest, and ongoing studies aim to develop strategies for patients affected by *L*-2HG efficiency.

### 2.5 SLC25A1 protein

As aforementioned, impairment of specific enzyme activities typically yields only one enantiomeric form of 2HG in the cellular milieu, thereby altering wild-type functionality and amplifying pathogenic processes. In contrast, disruption of a particular mitochondrial carrier protein, solute carrier family 25 member 1 (*SLC*25A1), is distinctive, as its mutation leads to an increase of a racemic mixture of both *D*-2HG and *L*-2HG, thereby exacerbating disease activity to a greater extent than other alterations. Elevated levels of both enantiomers of 2HG in human body fluids have been strongly associated with mutations in *SLC*25A1, a member of the mitochondrial carrier protein family (*SLC*25), which comprises 53 human isoforms (*SLC*25A1-*SLC*25A53) (76). Specifically, *SLC*25A1 encodes a citrate carrier protein located in the inner mitochondrial membrane, consisting of 311 amino acids with a molecular mass of 34.013 kDa, and is mapped to chromosome 22q11.2. Its principal role is the regulation of citrate flux between the mitochondria and cytoplasm. Citrate exported by *SLC*25A1 serves not only as an intermediate in the TCA cycle but also as a precursor for fatty acid and sterol synthesis, glycolysis regulation, MAL production, and energy metabolism, as well as for epigenetic modifications via histone acetylation and other physiopathological processes (120). Mutations in *SLC*25A1 are reported as homozygous, heterozygous, hemizygous, and frameshift alleles, which are strongly linked to the overproduction of both *D* and *L*-2HG *(*121–124). More than 24 distinct mutations have been identified, several of which directly impair protein binding sites, reducing *SLC*25A1 activity and availability. Notably, the D69Y and R247Q (homozygous) and R210X and V49M (heterozygous) mutations markedly suppress *SLC*25A1 function, resulting in mitochondrial dysfunction, reduced brain volume, and cardiac impairment (123, 125). These abnormalities manifest clinically as myopathy, encephalopathy, and neuropathy (76).

Disruption of *SLC*25A1 function severely affects the synthesis of TCA intermediates, compromising ATP production, amino acid biosynthesis, fatty acid metabolism, and other essential biochemical processes. Clinical data reveal that impaired *SLC*25A1 activity leads to elevated urinary excretion of TCA intermediates, including α-KG, succinate, fumarate, MAL, and 2HG (126). Fibroblast-based analyses of wild-type *SLC*25A1 show increased citrate efflux and a concomitant reduction in intracellular 2HG, underscoring its protective metabolic role. Conversely, mutations amplify 2HG by diminishing mitochondrial citrate availability, thereby disrupting glycolysis and reducing acetyl-CoA formation (122). Given that acetyl-CoA is central to lipid, sterol, and fatty acid biosynthesis, as well as energy metabolism (127). The depletion of acetyl-CoA contributes to widespread metabolic malfunctions and sustained 2HG elevation. Experimental study of zebrafish models supports that the inactive *SLC*25A1 proteins exhibit brain shrinkage, mitochondrial loss, and cardiac dysfunction (123). Elevated 2HG levels resulting from *SLC*25A1 impairment are closely linked with tumorigenesis, mainly through inhibition of tumor suppressor proteins and subsequent oncogene activation (128). However, the precise mechanistic pathway by which *SLC*25A1 deregulation facilitates 2HG magnification and oncogenic transformation remains incompletely understood, warranting further investigation.

## 3 The pathogenic role of 2HG in cellular functions

**Figure 2:**
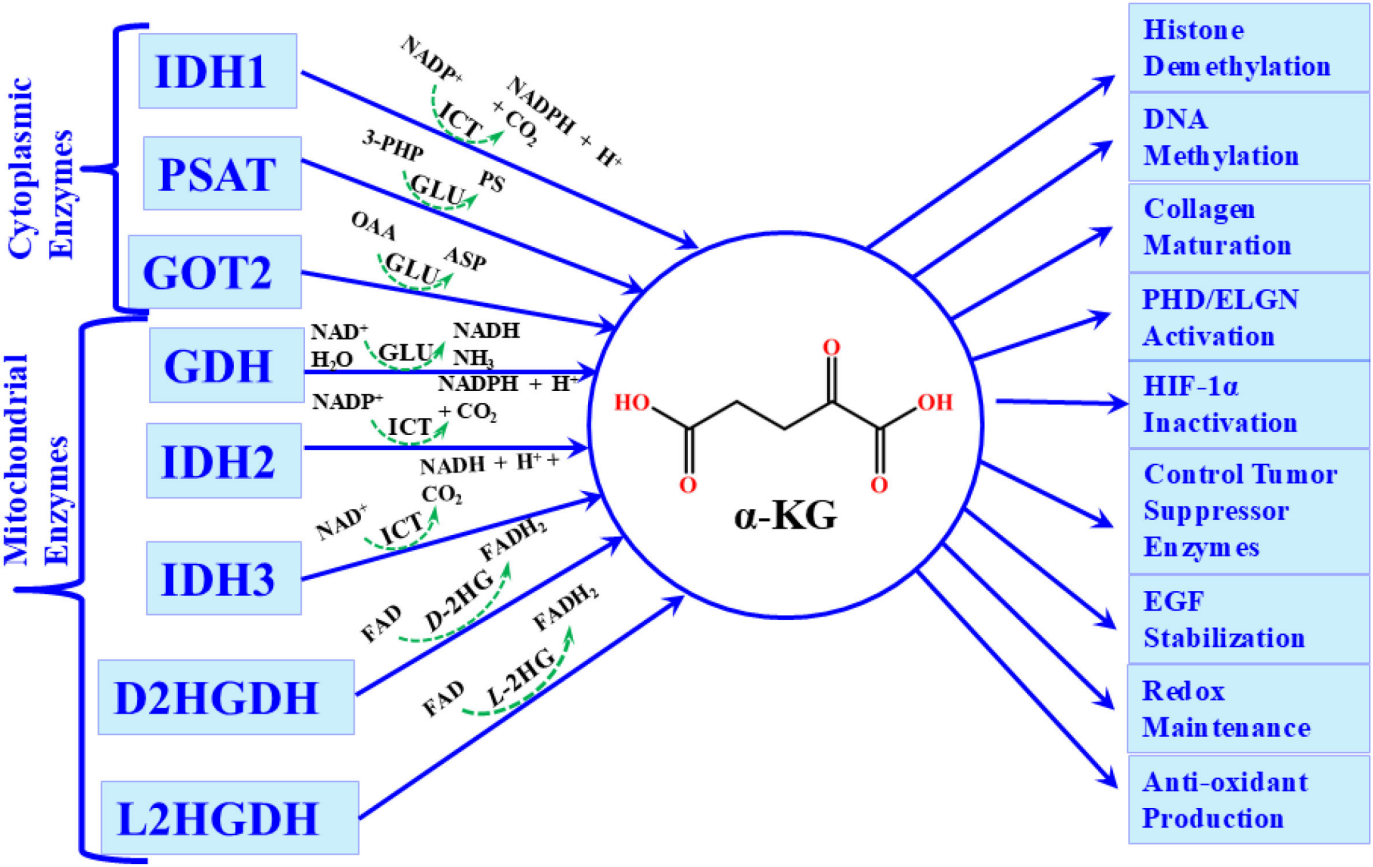
Pictorial representation of the cellular synthesis of α-KG and its functional importance in various biochemical reactions.

The fundamental pathogenic role of 2HG is its ability to steal the α-KG metabolic pathway, particularly α-KG-dependent dioxygenase reactions, thereby enhancing abnormal cellular activities. Structurally, 2HG closely resembles α-KG, differing only by the substitution of a hydroxyl group at C2 in place of the keto group present in α-KG. Due to this structural similarity, 2HG interrupts the α-KG-dependent dioxygenases. α-KG is an essential metabolite with critical roles in numerous cellular processes. α-KG-dependent dioxygenases catalyze reactions that regulate the modification of DNA, RNA, proteins, and lipids, including epigenetic alterations that control gene expression (129). Among these processes, histone methylation and demethylation are particularly significant, as they govern the activation or silencing of DNA sequences (130). These mechanisms influence transcription, translation, chromatin packaging, and DNA repair. For example, histone tails H3 and H4 are frequently methylated at lysine and arginine residues to modulate chromatin conformation and thereby alter gene expression (131). The Jumonji (Jmj) domain is a key catalytic module in histone demethylases, comprising N-methyl lysine and N-methyl arginine demethylases, which function as α-KG-dependent oxygenases to remove methyl groups and regulate gene activity (132). Inhibition of α-KG availability therefore profoundly disrupts histone demethylation, disturbing gene “switching” processes and directly linking epigenetic deregulation to tumorigenesis (133). Similarly, α-KG-dependent ten-eleven translocation (TET) dioxygenases drive DNA demethylation at CpG islands by converting 5-methylcytosine (5mC) into 5-hydroxymethylcytosine (5hmC), which is subsequently oxidized to 5-formylcytosine (5fC) and 5-carboxylcytosine (5caC) (134). Loss of α-KG availability or inhibition by 2HG disrupts TET activity, leading to aberrant DNA methylation, transcriptional misregulation, and enhanced oncogenesis (129). The deregulation of TET enzymes also affects the pluripotency and differentiation of embryonic stem cells, with oncogenic signaling interfering with lineage-specific differentiation programs (135). More broadly, eight groups of α-KG-dependent dioxygenases regulate the hydroxylation of proteins (e.g., collagen and epidermal growth factor (EGF) domains), are interlinked with histone demethylation via Jmj domains, and are oxygen-sensing under hypoxia. Collectively, these enzymes compose protein stabilization, transcriptional control, ribosome function, epigenetic modifications, angiogenesis, metabolic homeostasis, ROS control, lipid biosynthesis, and mitochondrial energetics (136) (**Figure 2**).

**Figure 3:**
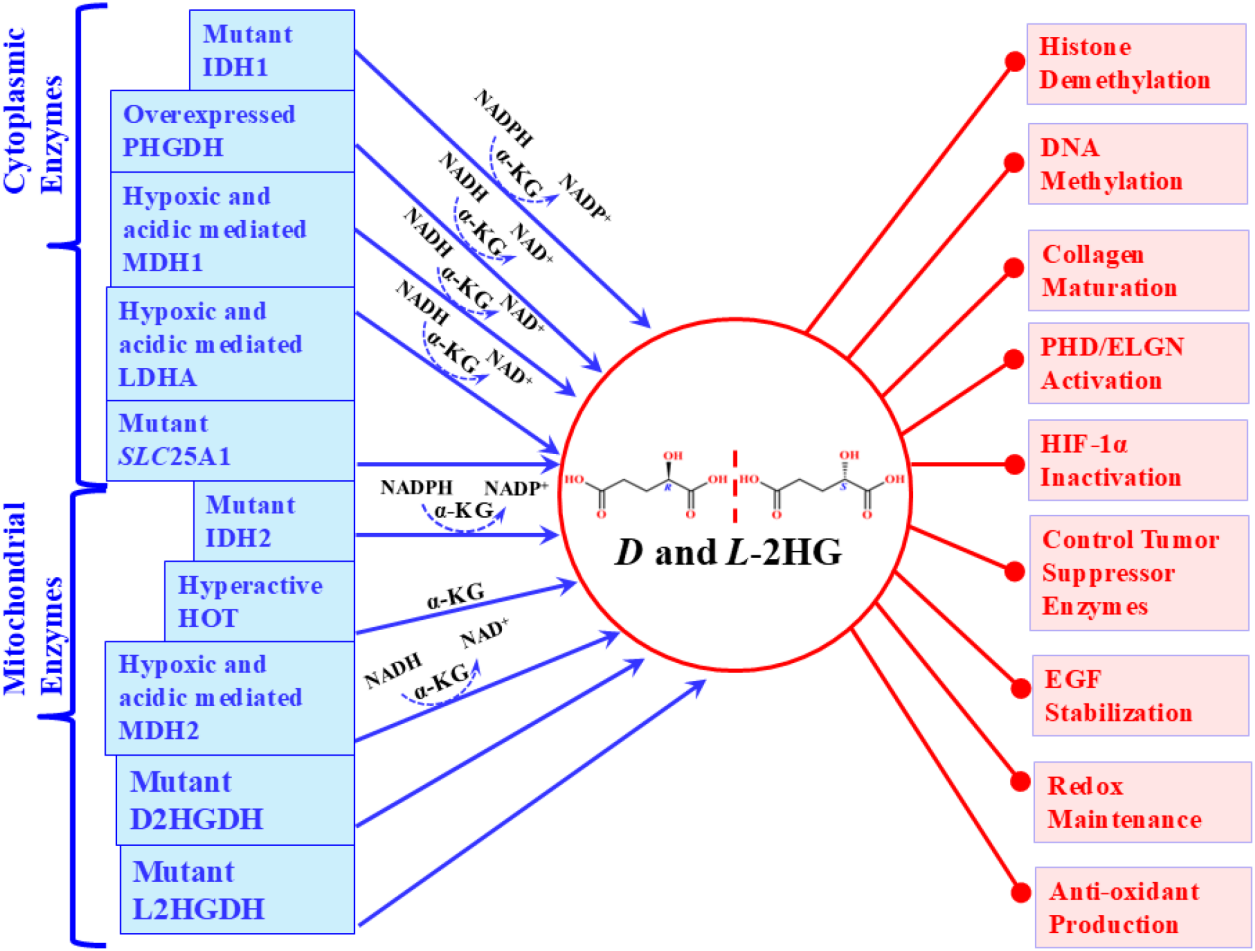
Pictorial representation of cellular synthesis and multiple biochemical pathogenic importance of *D* and *L*-2HG.

2HG has been experimentally demonstrated to divert multiple α-KG-dependent dioxygenases, with a particularly marked effect on histone demethylases. Elevated 2HG increases histone methylation, particularly H3K4me3, and modifies residues such as H3K9, H3K36, and H3K79 (137). Mutations in IDH1 and IDH2 elevate 2HG levels, inducing hypermethylation at H3K9 and H3K27, thereby blocking histone demethylation and promoting oncogenic epigenetic alterations (138). Furthermore, 2HG antagonizes α-KG at Jmj domain-containing proteins, potently inhibiting demethylases such as JMJD2A, JMJD2C, and FBXL11, with half-maximal inhibitory concentrations (IC50) ≥100 µM (139). This epigenetic reprogramming extends to DNA, where 2HG heightening is strongly linked to the glioma CpG island methylator phenotype (G-CIMP) observed in grade II/III gliomas and glioblastomas. G-CIMP arises through TET enzyme inactivation, which suppresses the demethylation of 5mC to 5hmC, leading to DNA hypermethylation, transcriptional silencing, and oncogene activation (140). Studies demonstrate that 2HG reduces 5hmC levels in human embryonic kidney HEK293-T cells by inhibiting TET1 and TET2 (141). Clinically, 2HG overproduction in breast cancers has been associated with extensive histone and DNA hypermethylation. The *MYC* knockdown reduces 2HG levels and diminishes tumorigenicity, underscoring the link between *MYC* expression, 2HG widening, and epigenetic deregulation (51)(**Figure 3**).

**Figure 4:**
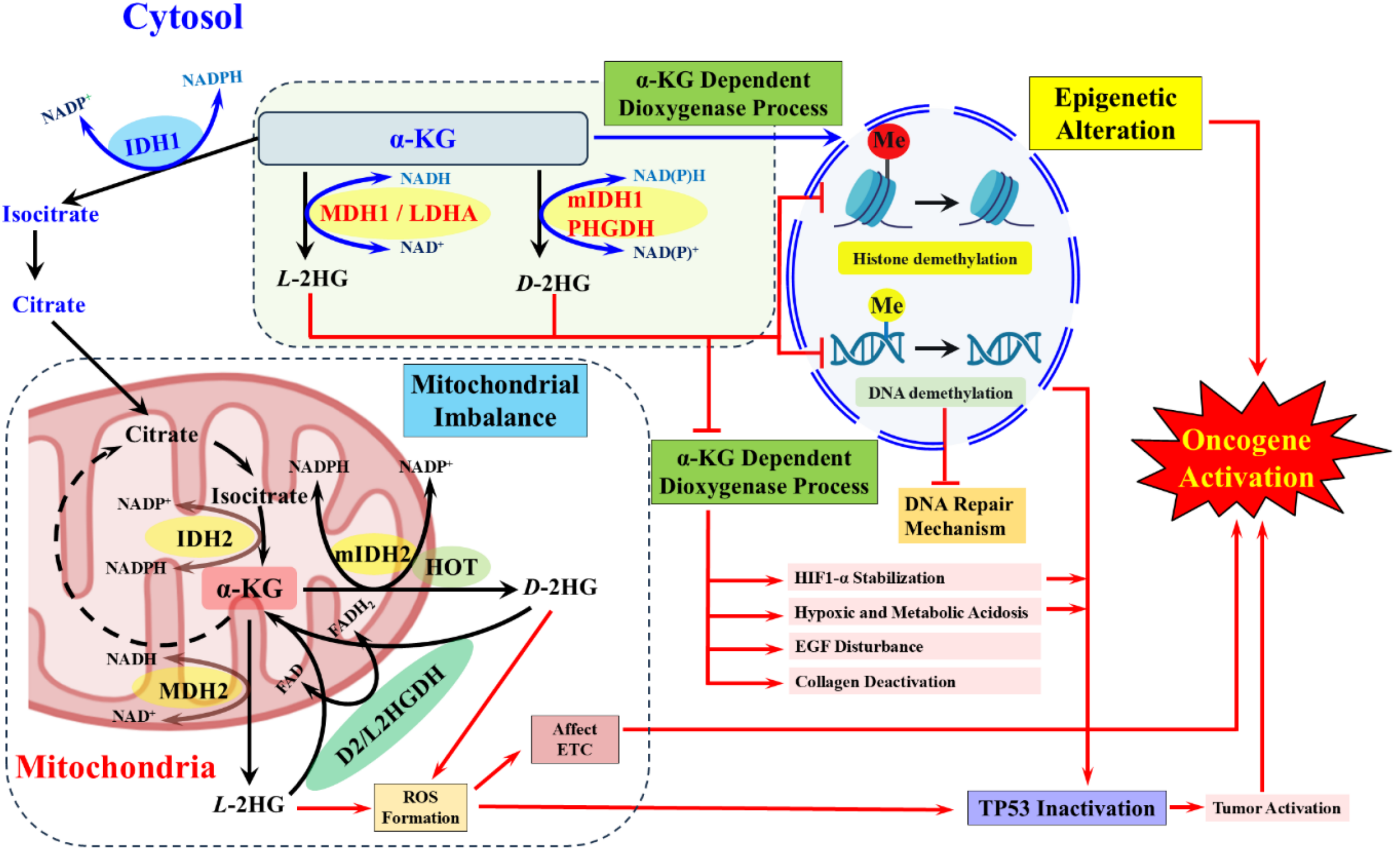
The core mechanistic pathway of 2HG in cytosol and mitochondria to affect the epigenetic alteration, DNA repair mechanism, inhibit α-KG-dependent dioxygenase process and affect the ETC via the ROS accumulation. This collective abnormalities enhance oncogene activation along with the inactivation of TP53.

An elevated level of 2HG can broadly affect TP53 activity, which is essential for regulating genomic stability and controlling cellular stress. Clinically, elevated levels of 2HG disrupt α-KG-dependent dioxygenases, leading to epigenetic modifications and transcriptional regulation of TP53 and its downstream pathway. The α-KG-dependent dioxygenases, chromatin alteration, and 5hmC stimulate TP53 activity, and elevated levels of 2HG divert this process towards tumor initiation (142). In addition, 2HG-arbitrated metabolic abnormalities endorse ROS hyper, enhance the metabolic acidity and hypoxic signaling, which can directly alter the TP53 (**Figure 4**) stability and activity (143, 144). The pathogenic mechanism of 2HG largely impairs the TP53 downstream pathway, thereby enhancing tumor progression and cellular stress response by inhibiting α-KG-dependent dioxygenase activity. Accretion of 2HG has also been linked with therapeutic resistance across numerous cancers. Fundamentally, 2HG affects α-KG-dependent dioxygenases, including TET-mediated DNA demethylases and Jmj-domain histone demethylases, catalyzing extensive epigenetic reprogramming that alters the expression of genes associated with drug sensitivity and cell survival (140, 145).

In addition, 2HG facilitates metabolic rewiring, endorses redox adaptation, and augments cancer cell survival under chemotherapeutic stress (146). Beyond epigenetics, boosting 2HG affects HIF-1α stabilization, a pivotal transcription factor in the process of oncogenesis. HIF-1α regulates tissue oxygenation, angiogenesis, and vascularization through targets such as vascular endothelial growth factor (VEGF) and erythropoietin (EPO), which are essential for red blood cell development, growth, and differentiation. Elevated 2HG rewire α-KG-dependent dioxygenases involved in HIF-1α hydroxylation and degradation, thereby stabilizing HIF-1α (69). Increased HIF-1α levels further promote ROS increment, cellular damage, and cancer progression (68). Prominent evidence suggests that the uplifted level of 2HG stimulates the tumor microenvironment by sustaining HIF-1α signaling and modifying immune cell signals, thereby promoting the fight against earmark therapies and immunotherapies (147). The 2HG performs as a pathogenic antagonist of α-KG, competitively inhibiting dioxygenases that regulate epigenetic modifications, DNA repair, and hypoxia signaling (140) to act as a metabolic driver. This mechanism broadly influences therapy resistance to alter the metabolic pathway to promote cancer cell proliferation. These disruptions collectively drive oncogenesis, linking 2HG escalation with both genomic instability and metabolic anomalies.

## 4 Conclusion and outlook

Extensive experimental investigations strongly suggest that metabolic alterations are a defining signature of cancers (3). Identifying oncometabolites has become a cornerstone of cancer research, and both enantiomers of 2HG have emerged as critical players. Derived from deregulated enzymatic reactions, 2HG accumulates to pathogenic levels and contributes significantly to malignant tumorigenesis, thereby establishing its role as a bona fide oncometabolite (13, 20). Mainly, the 2HG can act chiefly as a permissive modifier, influencing tumor progression by emphasizing metabolic rewiring, redox imbalance, and hypoxia signaling rather than helping as the originating oncogenic event. These remarks highlight the context-dependent role of 2HG in cancer metabolism and underline the importance of distinguishing between driver and modulatory functions of oncometabolites. Mechanistically, 2HG exerts its pathogenicity by antagonizing α-KG and competitively inhibiting α-KG-dependent dioxygenases, thereby driving oncogenic pathways (21, 129).

**Figure 5:**
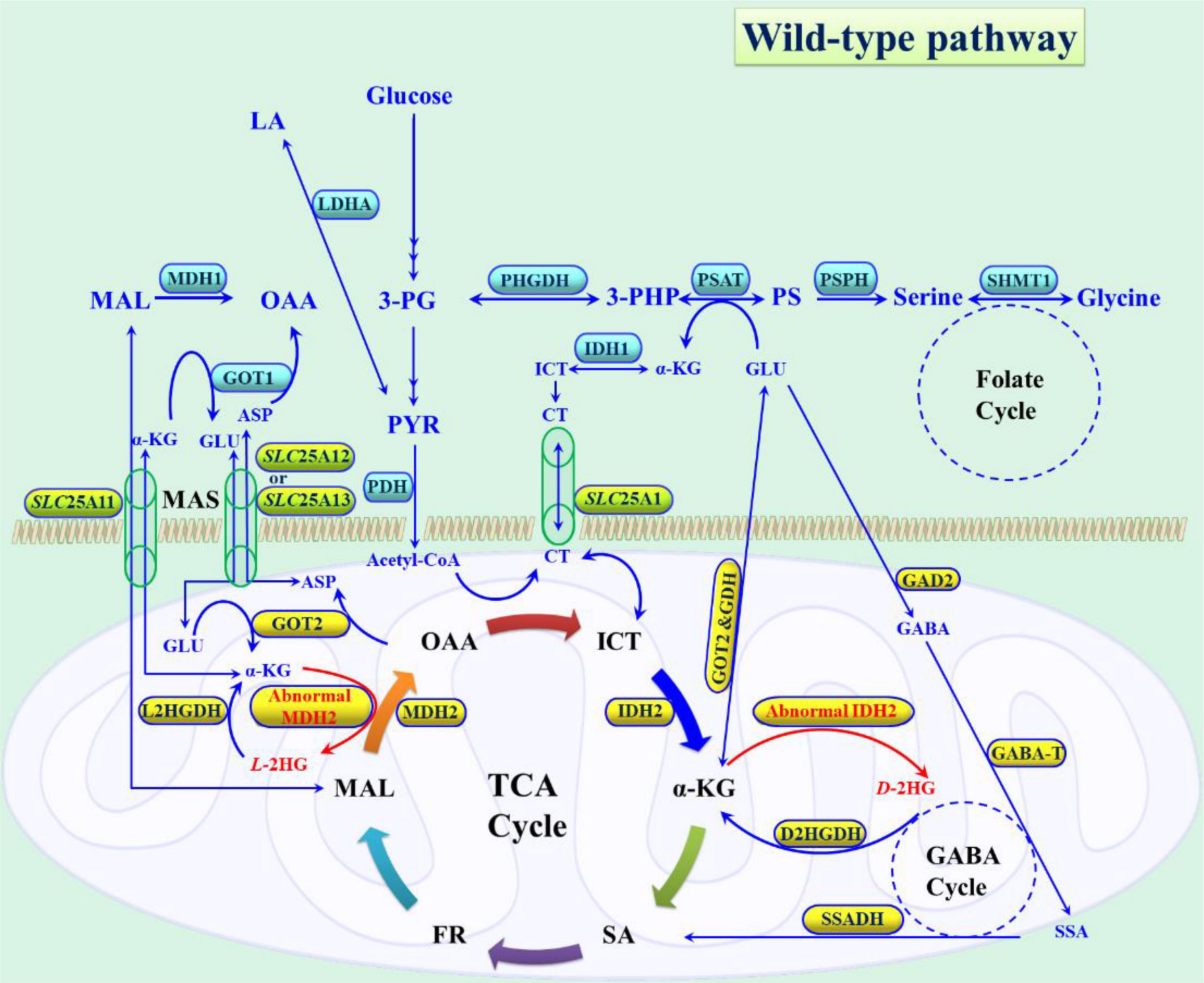
Pictorial representation of the wild-type pathway of enzymatic reactions in the cytoplasm and mitochondrial compartments.

**Figure 6:**
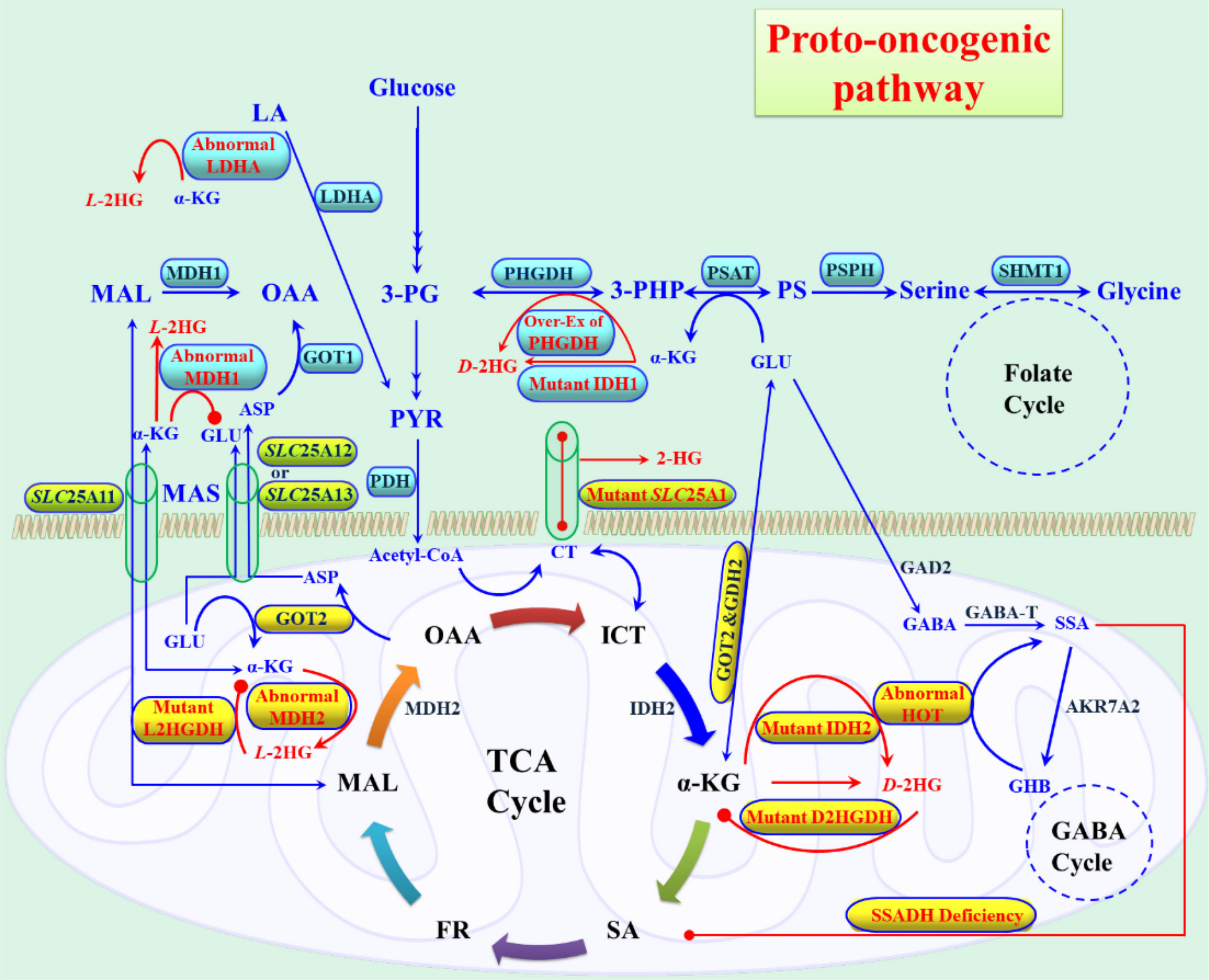
Pictorial representation of the proto-oncogenic pathway of deregulated enzymatic reactions in the cytoplasm and mitochondrial compartment.

In the cytosol, mutant IDH1 and overexpressed PHGDH predominantly generate *D*-2HG, while hypoxia and metabolic acidosis-mediated LDHA and MDH1 produce *L*-2HG. In the mitochondria, mutant IDH2, aberrant HOT activity, and impaired D2HGDH generate *D*-2HG, whereas mutant L2HGDH, along with hypoxia and metabolic acidosis-driven MDH2, enhance *L*-2HG formation. Importantly, in the cytosol, clearance of 2HG is inefficient due to the limited availability of repair enzymes (D2HGDH and L2HGDH), whereas mitochondrial 2HG can be partially converted back to α-KG (21). Only trace cytosolic 2HG is excreted in urine, and the remainder profoundly modifies cellular functions. At millimolar concentrations, mitochondrial 2HG promotes mitochondrial dysfunction, ROS production, enzyme impairment, and oncogene activation (32, 71). For example, IDH2 mutations drive *D*-2HG formation, impair D2HGDH, and disrupt PHD/EGLN activity, thereby stabilizing HIF-1α and creating a pseudo-hypoxic state (21). This pseudo-hypoxia enhances metabolic acidosis, which, in turn, protonates α-KG and increases HOT hyperactivity, resulting in up to 10 to 15-fold higher *D*-2HG concentrations (54). Protonated α-KG also becomes more reactive, favoring its reduction by MDH2 to *L*-2HG over *L*-MAL. Notably, *L*-2HG is ≥17-fold more potent than *D*-2HG in inhibiting α-KG-dependent dioxygenases, impairing L2HGDH activity, and stabilizing HIF-1α, thereby reinforcing hypoxic and acidic cellular conditions (98). The interplay between hypoxia and acidosis is cyclical, where metabolic acidosis stabilizes HIF-1α even in normoxia, while hypoxia enhances acidosis (69). Stable HIF-1α transcriptionally modulates numerous enzymes, including NNT, reducing mitochondrial NADPH availability. This limits α-KG to ICT conversion, thereby decreasing citrate supply (77). Diminished citrate availability impairs *SLC*25A1 activity, leading to a rise in racemic 2HG (122). Excess α-KG from IDH2 mutations and GLU metabolism is also shuttled into the cytosol via *SLC*25A11, where hypoxic and acidic conditions further elevate 2HG levels (148). These conditions reinforce the Warburg effect, stimulating mutant IDH1 (149), overexpressed PHGDH (64), upregulated LDHA (98), and MDH1 (96), thereby amplifying 2HG production (**Figures 5**, **6**).

**Figure 7:**
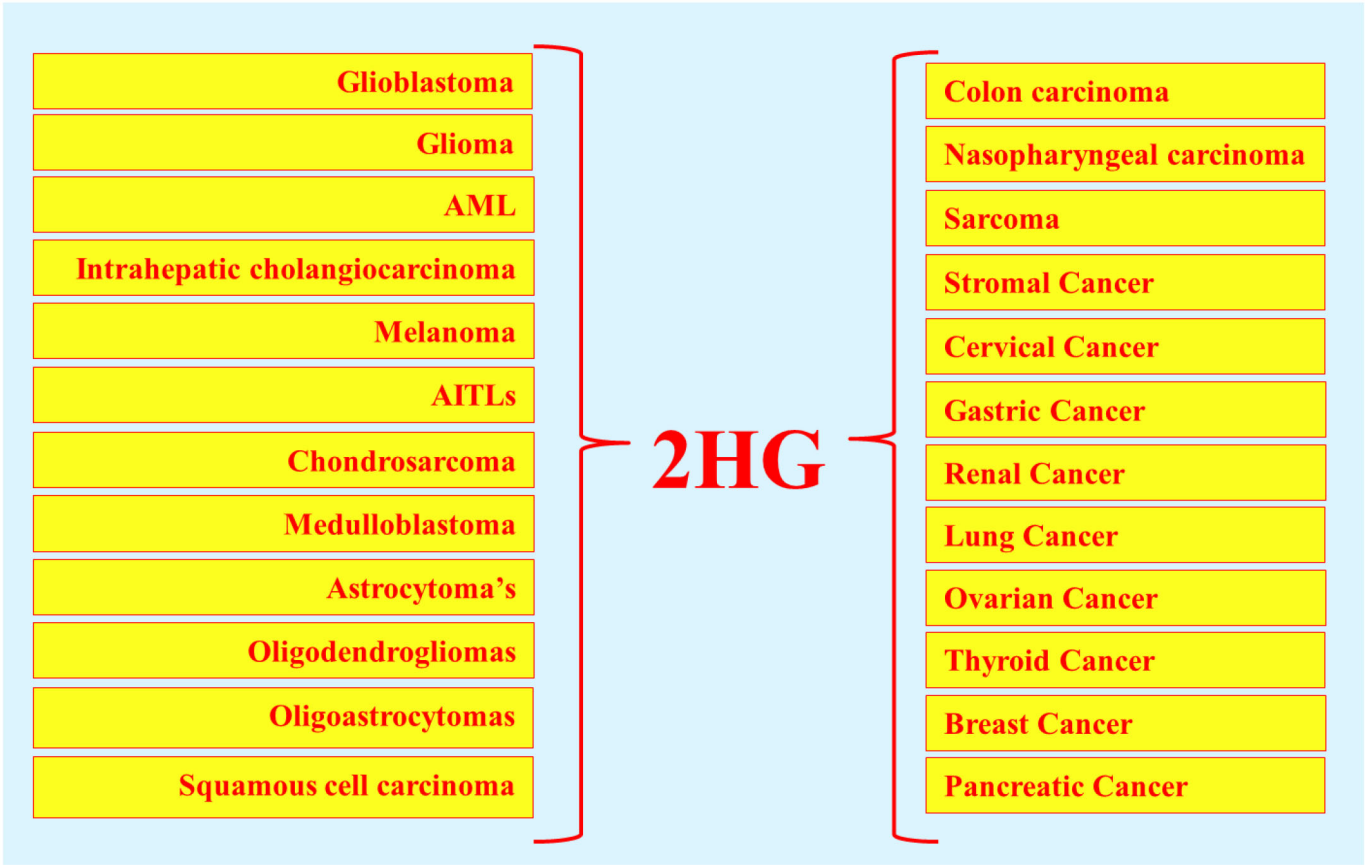
Pictorial representation of cancers and other disorders associated with oncometabolite 2HG.

The complete core mechanistic pathway of 2HG formation clearly reveals how oncogene activation affects cellular processes across different biological compartments (**Figure 4**). This vicious cycle perpetuates epigenetic deregulation via histone and DNA hypermethylation (51), impairs collagen maturation and EGF signaling (136), and drives the progression of diverse cancers, including glioma, glioblastoma, acute myeloid leukemia (AML), intrahepatic cholangiocarcinoma, melanoma, angioimmunoblastic T-cell lymphoma (AITL), chondrosarcoma, sporadically in melanoma, medulloblastoma, astrocytomas, hepatocellular, oligodendrogliomas, oligoastrocytomas, squamous cell carcinoma, colon carcinoma, nasopharyngeal carcinoma, sarcoma, stromal tumors, cervical cancer, gastric cancer, renal carcinoma, lung cancer, ovarian cancer, thyroid cancer, breast cancer, and pancreatic cancer (24, 39, 40, 50, 57, 61, 62) (**Figure 7**).

Apart from the mechanistic role in tumor metabolism, 2HG has arisen as a validated biomarker for various malignancies to initiate cancer progression. Advances in metabolomic analytical techniques, such as magnetic resonance spectroscopy (MRS) and mass spectrometry (MS), now enable the detection of 2HG in tumor tissues and patient biofluids. Numerous clinical studies have explored its diagnostic, prognostic, and therapeutic potential (shown in **Table 1**).

**Table 1:** Clinical evidence for supporting 2HG as a biomarker in various cancers.

Cancer type	Enzyme type	Enantiomer detected	Detection method	Sample type	Clinical significance	Sensitivity/specificity
Glioma	IDH 1/2	*D*-2HG	MRS	Tumor tissue	Non-invasive marker of IDH mutation	High sensitivity (150)
Glioblastomas	IDH 1/2	*D*-2HG	Liquid chromatography-mass spectrometry (LC-MS)	Cerebrospinal Fluid (CSF)	Serve as a liquid biopsy biomarker	Diagnostic Validation (151)
AML	IDH 1/2	*D*-2HG	MS	Serum, urine, bone marrow	Diagnostic marker of IDH mutation and therapy monitoring	High specificity for IDH mutation (152)
Intrahepatic cholangiocarcinoma	IDH 1/2	*D*-2HG	LC-MS	Serum	Surrogate biomarker IDH1 and 2 mutation	High sensitivity and specificity (153)
Breast cancer and Melanoma	PHGDH	*D*-2HG	LC-MS	Cell culture and *in-vitro* enzymatic assays	Connected with metabolic reprogramming in cancers	Not reported (64)
AITLs	IDH 2	*D*-2HG	Gene sequencing and mutation analysis	Tumor tissue	Associated with epigenetic alteration	Not reported (154)
Chondrosarcoma	IDH 1/2	*D*-2HG	LC/MS and MRS	Tumor tissue	Non-invasive marker of IDH mutation	High sensitivity (155)
Neurometabolic disorders, including medulloblastoma, astrocytomas and oligodendrogliomas	L2HGDH	*L-*2HG	Gas chromatography-mass spectrometry (GC-MS)	Urine and CSF	Diagnostic metabolic biomarker for neurometabolic disease	Not reported (156)
Head and neck squamous cell carcinoma	Not specified	*D*-2HG	LC/MS and GC/MS	Saliva samples	Non-invasive biomarker for oral cancers	Not reported (157)
Colorectal cancer	Not specified	*D*-2HG	LC/MS	Tumor tissue	Strongly correlated with tumor metastasis in colorectal cancers	Not reported (158)
Lung, colorectal, nasopharyngeal, and renal cell carcinoma	IDH1, IDH2 and L2HGDH	Both *D* and *L-*2HG	LC/MS	Serum, urine, and plasma	Potential recognition for 2HG enantiomers	High sensitivity (159)
Sarcoma	IDH 1/2	*D*-2HG	LC/MS	Tumor tissue	Potently connected with IDH1 and 2 mutations in various sarcomas	Not reported (160)
AML and stromal	IDH 1/2	*D*-2HG	LC/MS	Cell culture models	Enhancing NF-κB signaling to promote stromal tumors in AML	Not reported (161)
Gastric cancer	IDH 1/2	*D*-2HG	LC/MS	Plasma and blood samples	Non-invasive biomarker for IDH mutants in cholangiocarcinoma	High sensitivity and specificity (162)
Ovarian cancer	PHGDH	*D*-2HG	Metabolomic analysis	Cancer cell lines	Upregulation of PHGDH in metabolic abnormalities and drug resistance	Not reported (163)
Thyroid cancer	Not specified	Both *D* and *L-*2HG	LC/MS	Tumor tissue	Metabolic biomarker allied with thyroid cancer	Not reported (164)
Breast cancer	ADHFE1	*D*-2HG	LC-MS	Tumor tissue	Linked with *MYC*-mediated metabolic changes	Investigational (54)
Renal and pancreatic tumors	LDHA and MDH	*L*-2HG	LC-MS	Cell lines and tumor tissues	Marker of hypoxia-driven metabolic fluctuation	Investigational (100)

Over the last decade, significant progress has been made toward therapeutic targeting of 2HG. Notably, the FDA approved inhibitors Ivosidenib (165) and Enasidenib (166) have shown efficacy in treating IDH mutant malignancies, particularly AML. However, many other inhibitors remain under clinical investigation, and the search for more effective agents is still ongoing (20, 167, 168).

## 5 Future perspectives

Despite noteworthy advances in understanding the metabolic and epigenetic role of 2HG, several key questions remain unanswered. Forthcoming studies should focus on elucidating the interaction between 2HG accumulation in the tumor microenvironment, particularly how elevated 2HG affects immune cell metabolism, immune dysfunction, and tumor-immune interactions. Interpreting these interactions may reveal novel mechanisms by which metabolic dysregulation promotes immune evasion in cancer. Another significant research direction is the identification of IDH-independent mechanisms of 2HG formation. Advanced research specifies that enzymes such as PHGDH, LDHA, and MDH isoforms potentially generate 2HG under metabolic stress, hypoxia, and acidic pH. Pointing out these alternative pathways may offer new therapeutic opportunities, particularly in tumors that lack IDH mutations but still exhibit elevated 2HG levels. Finally, advances in metabolomics, isotope tracing, and single-cell metabolic profiling are anticipated to deliver deeper insights into the spatial and temporal dynamics of 2HG metabolism in tumors. Incorporating these technologies with a multi-omics tactic may accelerate the expansion of precision metabolic drug targeting of 2HG-driven oncogenic pathways.

## Glossary

ATP: Adenosine Triphosphate
LA: Lactate
PKM2: Embryonic pyruvate kinase isoform M2
ROS: Reactive Oxygen Species
PPP: Pentose phosphate pathway
PI3K: Phosphatidylinositol 3-kinase
mTOR: Mammalian target of rapamycin
TCA: Tricarboxylic acid
2HG: 2-hydroxyglutarate
IDHs: Isocitrate dehydrogenases
ICT: Isocitrate
GLU: Glutamate
NADP^+^: Nicotinamide adenine dinucleotide phosphate
α-KG: α-ketoglutarate
NAD^+^: Nicotinamide adenine dinucleotide
ADP: Adenine dinucleotide phosphate
PDH: Pyruvate dehydrogenase
TP53: Tumor suppressor protein 53
GBM: Glioblastoma
AML: Acute myeloid leukemia
AITL: Angioimmunoblastic T cell lymphoma
HOT: Hydroxyacid-oxoacid transhydrogenase
ADHFE1: Alcohol dehydrogenase iron-containing protein 1
GHB: γ-hydroxybutyrate
SSA: Succinic semialdehyde
D2HGDH: D-2-hydroxyglutarate dehydrogenase
EMT: Epithelial-mesenchymal transition
EMP: Embden-Meyerhof pathway
SSP: Serine synthesis pathway
PHGDH: Phosphoglycerate dehydrogenase
3-PG: 3-phosphoglycerate
3-PHP: 3-phosphohydroxypyruvate
PS: Phosphoserine
PSAT1: Phosphoserine aminotransferase
PSPH: Phosphoserine phosphate
ER: Estrogen receptor
HIF-1α: Hypoxia-inducible factor-1α
CNS: Central nervous system
HIF: Hypoxia-inducible factor
FIH: Factor inhibiting HIF
VHL: von Hippel-Lindau
HREs: Hypoxia response elements
GLUT: Glucose transporters
LDH: Lactate dehydrogenase
MDH: Malate dehydrogenase
PDK: Pyruvate dehydrogenase kinase
HK: Hexokinases
PGK1: Phosphoglycerate kinase 1
ALD: Aldolases
PYR: Pyruvate
MAL: Malate
NNT: Nicotinamide nucleotide transhydrogenase
OAA: Oxaloacetate
MAS: Malate-aspartate shuttle
GOT: Glutamic-oxaloacetic transaminases
L2HGDH: L-2-hydroxyglutarate dehydrogenase
MCT4: Monocarboxylate Transporter 4
CAIX: Carbonic Anhydrase IX
EPR: Endoplasmic Reticulum
ETDs: Electron transport defects
FAD: Flavin adenine dinucleotide
PNETs: Primitive neuroectodermal tumors
SLC25A1: Solute carrier family 25 member 1
Jmj: Jumonji
TET: Ten-eleven translocation
5mC: 5-methylcytosine
5hmC: 5-hydroxymethylcytosine
5fC: 5-formylcytosine
5caC: 5-carboxylcytosine
G-CIMP: Glioma CpG island methylator phenotype
VEGF: Vascular endothelial growth factor
EPO: Erythropoietin
EGF: Epidermal growth factor
ETC: Electron Transport Chain
MRS: Magnetic Resonance Spectroscopy
MS: Mass Spectrometry
CSF: Cerebrospinal Fluid
LC-MS: Liquid chromatography-mass spectrometry
GC-MS: Gas chromatography-mass spectrometry.
